# Habitat Availability and Heterogeneity and the Indo-Pacific Warm Pool as Predictors of Marine Species Richness in the Tropical Indo-Pacific

**DOI:** 10.1371/journal.pone.0056245

**Published:** 2013-02-15

**Authors:** Jonnell C. Sanciangco, Kent E. Carpenter, Peter J. Etnoyer, Fabio Moretzsohn

**Affiliations:** 1 Marine Biodiversity Unit/Global Marine Species Assessment, Global Species Programme, International Union for Conservation of Nature, Gland, Switzerland; 2 Department of Biological Sciences, Old Dominion University, Norfolk, Virginia, United States of America; 3 NOAA Center for Coastal Environmental Health and Biomolecular Research, Charleston, South Carolina, United States of America; 4 Harte Research Institute for Gulf of Mexico Studies, Texas A&M University-Corpus Christi, Corpus Christi, Texas, United States of America; Consiglio Nazionale delle Ricerche (CNR), Italy

## Abstract

Range overlap patterns were observed in a dataset of 10,446 expert-derived marine species distribution maps, including 8,295 coastal fishes, 1,212 invertebrates (crustaceans and molluscs), 820 reef-building corals, 50 seagrasses, and 69 mangroves. Distributions of tropical Indo-Pacific shore fishes revealed a concentration of species richness in the northern apex and central region of the Coral Triangle epicenter of marine biodiversity. This pattern was supported by distributions of invertebrates and habitat-forming primary producers. Habitat availability, heterogeneity, and sea surface temperatures were highly correlated with species richness across spatial grains ranging from 23,000 to 5,100,000 km^2^ with and without correction for autocorrelation. The consistent retention of habitat variables in our predictive models supports the area of refuge hypothesis which posits reduced extinction rates in the Coral Triangle. This does not preclude support for a center of origin hypothesis that suggests increased speciation in the region may contribute to species richness. In addition, consistent retention of sea surface temperatures in models suggests that available kinetic energy may also be an important factor in shaping patterns of marine species richness. Kinetic energy may hasten rates of both extinction and speciation. The position of the Indo-Pacific Warm Pool to the east of the Coral Triangle in central Oceania and a pattern of increasing species richness from this region into the central and northern parts of the Coral Triangle suggests peripheral speciation with enhanced survival in the cooler parts of the Coral Triangle that also have highly concentrated available habitat. These results indicate that conservation of habitat availability and heterogeneity is important to reduce extinction of marine species and that changes in sea surface temperatures may influence the evolutionary potential of the region.

## Introduction

Persistent questions remain regarding the origins of the uneven distribution of marine species richness across the tropical Indo-Pacific [1], despite numerous relevant ecological and biogeographical studies and an urgent need to improve conservation effort [2]. In particular, explanations for the Coral Triangle epicenter of marine biodiversity [3] have received more attention in the literature than any other topic in marine biogeography [4]. This area encompasses much of Indonesia, Malaysia, the Philippines, Papua New Guinea, the Solomon Islands, Timor L’Este, and Brunei. It is also referred to as the East Indies Triangle [5]–[7], the Indo-Malay-Philippine Archipelago [8], [9], and a variety of other names [1]. The term Indo-Australian Archipelago (e.g. [10], [11]) is also used frequently, though the Coral Triangle does not include Australia [3] and does include geological elements beyond Indonesia [12].

The tropical Indian and Pacific Oceans encompass about two thirds of the earth’s equatorial circumference and include two distinct marine zoogeographic regions [13], [14], the Eastern Tropical Pacific and the Indo-West Pacific. Their shore biotas are effectively separated by a pelagic eastern Pacific barrier, a vast expanse of open ocean that lacks shallow island stepping stones for dispersal [13], [15]. The Eastern Tropical Pacific has limited shelf area and coral reef development. Species in this region have primary biogeographic affinities in the Caribbean except perhaps for some reef-building corals [16]. The tropical Indo-West Pacific is the most biologically diverse marine region worldwide, and is also the largest marine biogeographic realm, extending longitudinally more than halfway around the world and through more than 60° of latitude [17] with several distinct provinces [14]. It is a tropical and subtropical region extending from the Indian Ocean (including the Red Sea and Persian Gulf) eastward to the central Pacific Ocean through Polynesia to Easter Island [13], [18]. The extent of shallow water area and the length of coastline in the Indo-Pacific is a result of a complex geological history in the region [19] that resulted in separate successive biodiversity hotspots (areas of exceptional species richness) that coincided with areas of tectonic collision [20]–[25]. This tectonic activity both created and maintained extensive and complex shallow-water habitat at different periods. The current biodiversity hotspot is the Coral Triangle where the Eurasian, Philippine, Pacific, and Australian plates collide and effectively closed off the Indo-Pacific equatorial seaway [26]. This constricted ocean circulation in the western Pacific and initiated the formation of the Indo-Pacific Warm Pool in the late Miocene [27]. This warmest open ocean area continues to influence global climate. The western part of the Coral Triangle [3] spatially coincides with the main part of the area of consistent peak temperatures above 29°C of the Indo-Pacific Warm Pool [28]–[30].

The observation that peaks in marine species richness throughout geologic time follow changing concentrations of available habitat is an extension of the area of refuge hypothesis, one of the numerous hypotheses invoked to explain the current epicenter of marine biodiversity in the Coral Triangle [1], [8], [31]. The area of refuge hypothesis [32] suggests that species richness mainly depends on the extent of shallow-water (photic and mesophotic) habitat available over geologic time to consistently provide niches and effectively reduce rates of extinction. This hypothesis also relates to positive species-area relationships, a long-standing paradigm in ecology [33]. The Coral Triangle currently has the greatest concentration of tropical shallow water habitat on Earth, encompassing highly diverse and extensive areas of coral reefs, mangroves, seagrass beds, estuaries, and soft-sediment habitats [6], [9], [34], [35]. The Coral Triangle also has the longest coastline in the Indo-Pacific [26], [36] and extensive shallow water area that contributes geographic complexity which ultimately leads to species diversification [25]. The extent and diversity of habitat appears to correspond closely with species-area and species-habitat diversity relationships for this region [1].

In addition to area of refuge, other hypotheses used to explain the current epicenter of marine biodiversity can be summarized as: 1) center of origin [17], [22], [37]–[43]; 2) area of overlap of Indian and Pacific Ocean biotas [6], [44]; 3) area of accumulation of peripherally-originating species [45], [46]; 4) tectonic integration of biotas [9], [47]; and, 5) available energy [48]. The available energy hypothesis suggests that more energy can support more species (less partitioning of available energy) or higher temperatures promote faster population turnover rates and hence faster rates of speciation and extinction. Evidence exists to support many of these proposed hypotheses suggesting a combination of factors promote species richness in the Coral Triangle [6], [8], [9], [20], [35], [49]–[51].

A neutral hypothesis has also been tested relating to the mid-domain effect [52], [53]. This effect states that the geometric factor of range size causes species to randomly accumulate at the center of a bounded domain. Although the Coral Triangle epicenter of diversity is at the approximate center of an Indo-Pacific domain, non-random predictors such as habitat area influence on species richness are more important than the mid-domain effect in shaping diversity gradients in the region [42], [52], [53]. More generally, a review of a large number of published studies [54] concluded “observed patterns of species richness are not consistent with the mid-domain hypothesis.” Despite the potential utility of a mid-domain model in describing species richness gradients, there are other potential null models and the choices and underlying assumptions of each of these models are still a subject of considerable debate [55], [56]. The mid-domain effect was not tested in a global analysis of marine biodiversity patterns because of the subjective nature of mid-domain delineation [57]. The same constraint confounds its inclusion in an Indo-Pacific study. It is questionable to test a single mid-domain across two distinct regions such as the tropical eastern Pacific and tropical Indo-West Pacific because of the intervening east Pacific barrier. Separate mid-domains or an inclusive mid-domain for the provinces within the tropical Indo-West Pacific is also questionable. Due to these numerous constraints and the fact that mid-domains are not generally supported [54], a mid-domain model was not considered in our hypothesis-testing. Instead, we focused on hypotheses relating to habitat availability and available energy as these were best supported in a global study of species richness [57].

**Figure 1:**
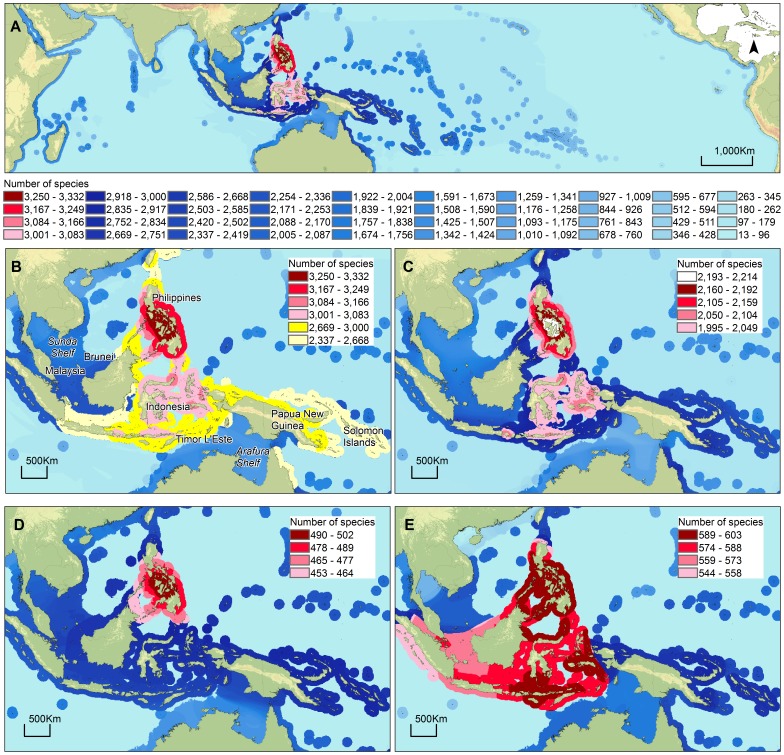
Patterns of species richness from range overlap raster data from 10,446 species. Each change in color represents an increase or decrease of 82 species (40 total classes or a 2.5% change per class). (A) Pattern of species distribution in the entire Indo-Pacific region. The top 10% for the highest species richness is found in the Coral Triangle (marked in red, pink, and yellow in panel B, with decreasing increments of species richness indicated by lighter shades), and the remaining decreasing increments of total species richness are indicated by lighter shades of blue, (B) The top 10% (shades of red), 20% (dark yellow) and 30% (light yellow) of concentration of species is in the Coral Triangle, with Philippines as the epicenter, (C) All fishes showing the top 1% of species richness (white); (D) Molluscs and crustaceans showing the top 10% of species richness (shades of red); (E) Habitat-forming species (corals, seagrasses, and mangroves) showing the top 10% of species richness (shades of red).

The area of refuge hypothesis was primarily formulated as an alternative to the well-established center of origin hypothesis which heavily relies on assumptions about dispersal [32] that presumably gave rise to the diminishing pattern of species richness with distance from the epicenter (Figure 1A). The area of refuge hypothesis is also founded in a long-held axiom in ecology, that larger areas hold more species than smaller areas [33]. Stehli and Wells (1971) [37], in the first depiction of the bull’s-eye pattern of species richness of coral genera in the Indo-West Pacific, invoked a center of origin hypothesis but also suggested that available area of coral habitat was important but that it “cannot yet be quantified.” In what is now called the area of refuge hypothesis [8], [9], [31], McCoy and Heck (1976) [32] first suggested that species-area relationships and extent of area available for refuge from extinction are responsible for tropical marine biogeographic pattern. They also invoke an eclectic approach in which the center of diversity in the Indo-West Pacific is accumulating species and that the extensive shoreline in this area allows for isolation and diversification of species. However, explicit in the area of refuge hypothesis as originally described by McCoy and Heck (1976) [32] is that the Coral Triangle has served as an area of lower extinction than the rate of either migration into or origination of species in the area over time. This basic concept is supported by other studies [11], [42], [58]. Evidence is accumulating that area of refuge, in terms of extensive and varied habitat, is an important factor in explaining species richness in the Indo-West Pacific [10], [53]. Many taxa also show lowered extinction risk that correlates with the extensive and diverse habitats of the Coral Triangle [42], [59], [60].

One component of the area of refuge hypothesis is habitat heterogeneity, which has long been considered important in shaping species diversity patterns [61], [62], [63]. A varied and complex habitat provides many different ways of exploiting environmental resources potentially catering to many species and promoting speciation when niches are not exploited by existing species. However, the accuracy of measuring the influence of habitat heterogeneity can depend on the index used, the spatial scale, and whether a ‘keystone structure’ has been identified that accurately reflects the species represented in the study [64]–[66]. For example, in a terrestrial system at small scales insect diversity may be dependent on presence of keystone vegetation types but at larger scales wetland presence may be the keystone structure [65]. There are many different keystone structures and grid sizes (scales) to choose from when testing predictors of species richness across the entire Indo-Pacific. In addition, alternative habitat variables may have different responses at varying scales. Coastline length has been proposed as a predictor of species richness [25], [32], [36], [57], and therefore can be hypothesized as a keystone structure to measure habitat availability and heterogeneity. In addition, available habitat in terms of extent and gradients of continental shelf area, reef area, seagrass bed area, and mangrove area can potentially influence species richness across the tropical Indo-Pacific [6], [67]–[69]. In previous explicit tests of species richness across the Indo-Pacific, available habitat and habitat heterogeneity have been lumped as either a conglomerate proxy in the form of coastline length [57] or different shallow area indices [53]. Here for the first time, we test the marine species richness predictive value of coastal length, available shelf area, and two different indices based on relative amounts of soft bottom, coral reef, seagrass beds, and mangrove swamps, in addition to available energy in the form of sea surface temperatures and net primary productivity.

It has long been established that there are latitudinal gradients in species richness with highest species richness in the tropics linked to multiple potential factors [61] although there are many exceptions to this gradient such as a subtropical peak in some oceanic tuna and shark species [57]. Explanations of this latitudinal gradient are that available kinetic energy [70] or patterns of primary productivity [66], [71], [72] influence rates of evolution. In the marine realm, sea surface temperature (SST) is most consistently linked to patterns of species richness on a global basis [57]. However, this pattern is weak within the tropics [71] and the energy hypothesis is specifically considered of insignificant predictive value for species richness in the tropical Indo-Pacific [53]. Indeed, in Rosen’s (1988) [31] review of biogeography of reef corals that largely addressed the concentration of species in the Coral Triangle, none of the 13 hypotheses he considered invoked an energy hypothesis. Frasier and Currie (1996) [48] went on to state “The most striking case that appears to contradict the species richness-energy hypothesis is that of coral reef organisms.” However, in their test of this statement they concluded that the best predictor of species richness was mean annual ocean temperature [48], but they did not directly address the relationship of SST to the position of the Indo-Pacific Warm Pool. Alternative hypotheses to explain marine species richness patterns such as the predictive value of environmental stress and stability have also been tested but only available habitat and available energy have consistent significant predictive value for marine species richness [48], [57]. In the present study, we reduced climate-related environmental effects influenced by latitudinal gradients by limiting the range of analysis to the tropics [66]. However, we retained average SST as a measure of available energy in our analyses because of its marked longitudinal variation in the Indo-Pacific as a result of the Indo-Pacific Warm Pool. Net primary productivity (NPP) was also retained as a predictor variable in our study as a possible factor related to the available energy hypothesis. Moreover, the nature, form, and structure of data quantifying taxonomic diversity and its ecological or evolutionary correlates change across scales [66]. Thus, we examined the effect on predictability of species richness relating to choice of scale, available energy, net primary productivity, available habitat, and habitat heterogeneity in the tropical Indo-Pacific.

## Methods

A number of different methods have been used to examine causes of the uneven distribution of marine species across the Indo-Pacific [1]. Recently, these include theoretical constructs based on existing studies [11], [21], [22], [41]; area cladograms [7], [73]; phylogenetic analyses and molecular clocks [25], [42], [60]; phylogeography (recently reviewed by Carpenter et al. 2011 [4]); and spatial analyses of distribution data [9], [10], [53], [57], [74]–[77]. Of those that used distribution data, Bellwood et al. (2005) [53] and Tittensor et al. (2010) [57] tested explicit alternative hypotheses and took into consideration autocorrelation, which violates one of the key assumptions in statistical analysis, that residuals are independent and identically distributed [78]. Bellwood et al. (2005) [53] tested hypotheses relating to the tropical Indo-Pacific but a large part of the data was based on a limited representation of reef fishes that shows an observed pattern of peak species richness very different from more complete studies [67]. The scope of the Tittensor et al. (2010) [57] study was global and used a large data set that includes cold-water species, and therefore, perhaps not a good test of patterns of species richness specifically for the tropical Indo-Pacific, although their fish distributions no doubt included a large proportion of tropical Indo-Pacific species.

### Spatial Range

The study range covers the entire Indian Ocean (including Red Sea and Persian Gulf) and the entire Pacific Ocean between 30°N and 30°S. All data layers were projected onto the World Cylindrical Equal Area coordinate system centered at 130° longitude.

### Grids and Grain Size

The study area was divided into equal grids of different horizontal resolutions (spatial grain) and the commonly-used Universal Transverse Mercator (UTM) grid to test the grid choice effects on relationships between predictors and species richness. Different orientations of centroid location were tested and found to have minimal effects (Text S1, Figures S1, S2, S3, S4, S5, and S6, Tables S1, S2) so therefore only the standard UTM centroid position results are presented here. The UTM is a grid-based system composed of 60 different zones globally. Although UTM grids are predisposed to distortion and area, the latitudinal range was limited to the tropics in this study, thereby limiting the effect of area distortion. Thus, each UTM grid measures about 617,000 km^2^/grid cell, except for the grids located along upper and lower edges of the study area which cover less area. To compare the effect of area distortion, we used, five different equal area grids: small (23,000 km^2^/grid cell), medium (92,000 km^2^/grid cell), large (368,000 km^2^/grid cell), extra large (1,470,000 km^2^/grid cell), and largest (5,100,000 km^2^/grid cell). The use of different grid sizes was not to test for the best possible grain but to test at which grain the predictors are likely to operate [79]. Some of the grids fall along the coastal areas and include land. This produces variable effective grid size in terms of available shallow water habitat and is a fundamental flaw with grids that has been dealt with in several ways, one of which is to combine adjacent grids [80]–[84]. Grid cells that contained nearly all land (these contained 85 to 99% of land) were removed from the analysis while others were combined with adjacent grid cells (about 6–10% in each grid size) to obtain water areas approximately equal to a single grid cell with 100% water (Figure S7; [82], [85]).

### Species Distribution Range Maps

The aim of this study was to test for effects of available nearshore habitat on species diversity, and therefore, relates to pelagic, benthic, or demersal species found primarily over continental or island shelves. The total number of species maps produced in this study was 10,446 (Table S3) of which 8,295 were coastal fishes, 1,212 are invertebrates (crustaceans and molluscs), and 939 habitat-forming species comprising of 820 reef-building corals, 50 seagrasses, and 69 mangroves. The set or range maps for coastal fishes (fishes that regularly occur over continental shelf), reef-building corals, seagrasses, and mangroves was comprehensive for these groups with the exception of those few species whose taxonomic validity or occurrence data was questionable. Generalized distribution range maps for these species were obtained from numerous expert-derived sources (Text S2) rather than relying on online databases that potentially suffer from a large proportion of inaccuracies [86]. These generalized species distribution maps were based on expert-verified occurrence localities that are used to bound extent of occurrence, or range, polygons. As such, they were not expected to relate to alpha diversity at highly local scales that were also influenced by habitat specificity, localized limitations to dispersal, and many other factors. Species distribution shapefiles were produced from standardized basemaps using ArcView 3.3 (Environmental Systems Research Institute, Redlands, CA). Two different types of basemaps were used, one for visualization of patterns of species richness and one for analyses relating species richness to independent variables (Figure S8). This study focuses on nearshore species, and therefore, the approximate maximum limit of continental and island shelves of 200 m isobath [87] was used for each species in analyses. However, this 200 m isobath sometimes occurs close to shore and in these areas, visualization of biodiversity patterns was difficult on the scale of the Indo-Pacific. Therefore, a biodiversity visualization basemap was created that consisted of a 100 km shoreline buffer if the 200 m depth contour was less than 100 km from shore or a 200 m depth contour limit if this occurred more than 100 km from shore. However, for pelagic species that occur over continental or island shelves and far from shore, extent of occurrence included open ocean inter-shelf areas within the species range. For analyses of species richness versus independent variables, each visualization basemap was cut to include only the area within the 200 m depth contour. All species distribution maps were converted into rasters of 10 km by 10 km cell size. The overlay raster tool in ArcGIS 9.3 (Environmental Systems Research Institute, Redlands, CA) was used to combine all the rasterized species distribution maps. This tool assigns a value for each cell that corresponds to the number of overlapping species ranges at the cell location, which was used to estimate species richness. The cell values of the combined rasterized maps were then classified into 40 classes of equal interval to show that each class corresponds to 2.5% of species composition.

### Habitat Availability and Heterogeneity

Using a Geographic Information System (GIS), we calculated the amount of continental shelf or shallow water area (SW) (km^2^) from a 200 m bathymetry layer processed from ETOPO1 (National Geophysics Data Center) raster data [88]. Land areas were erased from marine habitat data layers using World Vector Shoreline data. This is a standard product of the U.S. Defense Mapping Agency and is a digital data file at a nominal scale of 1∶250,000, containing the shorelines, international boundaries and country names of the world [89]. We also used the World Vector Shoreline data to generate the coastline length (km) by converting this to a line using conversion tools in ArcGIS 9.3. Coastline length (CL) was used here as a proxy for available nearshore habitat. To account for cases where grids contained no CL (offshore continental shelf area grid cells most common in small and medium size grids), 0.5 was added to all values of CL as a dummy variable.

We used coral reef, seagrass, and mangrove habitat GIS layers derived largely from atlases [90]–[92] provided by UNEP World Conservation Monitoring Centre to evaluate habitat heterogeneity. These map layers are high-resolution maps typically prepared from remotely-sensed data but in some cases mapped entirely from field observations. From these layers, we developed two indices – a habitat diversity index using area (HDIa) and a habitat diversity index using number of patches (HDIn). We calculated the habitat diversity indices using a modification of the Shannon-Weiner diversity index [93]. These indices essentially measure entropy and they will be highest when each habitat occupies the same amount of area in a grid cell. The HDIa was calculated from the values of the total area for each habitat including coral reefs (we assume that all hard bottom habitats in the tropics will have a varying degree of coral-reef biota associated with it), seagrass beds, mangrove forest, and soft-bottom areas for each grid. For HDIn, we substituted the measure of area with the number of patches of individual polygons of coral reefs, seagrasses, mangroves, and soft-bottom polygons within the 200 m bathymetry layers per grid cell. Coral reef, seagrass bed, and mangrove forest were composed of multiple independent polygons (i.e., patches) while the patches for soft-bottom 200 m bathymetry were a result of converting raster file (ETOPO1) into vector in ArcGIS 9.3. The number of patches of a particular habitat type may affect a variety of ecological process and often serves as an index of spatial heterogeneity [94]. The Shannon-Weiner Information Theory formula [93] was given as:
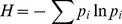
or



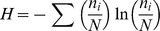
where *n_i_* = area (or number of patches) for each habitat and *N* = total habitat area (or total number of patches).

### Sea Surface Temperature and Net Primary Productivity

The SST layer was developed from the monthly long-term SST data derived from the National Oceanic and Atmospheric Administration Optimum Interpolation dataset. This product was constructed from two intermediate climatologies to produce a 1° resolution dataset with a 1961–1990 base period [95]. The NPP layer was generated from the standard monthly products available in monthly files. These products used the Vertically Generalized Productivity Model [96] as the standard algorithm, which estimates the productivity using a temperature-dependent description of chlorophyll-specific efficiency. Monthly NPP rasters from 2002–2010 were downloaded and then geoprocessed to get the average value. Both SST and NPP raster layers were converted into point vector shapefiles, which captured the values to be used in overlaying with different grid sizes. In a few cases (28 out of 1,390 cells for the smallest grid size and 3 out of 570 cells for the medium grid size), SST values were not available for certain grid cells and an estimated value was obtained by averaging values from neighboring cells.

### Statistical Analysis

Range overlap maps and environmental layers were combined to form grid matrices with the corresponding values of species richness, habitat availability (i.e. shallow water area, coastal length), two habitat heterogeneity indices (i.e. calculated from area and number of patches of coral reefs, seagrass beds, mangrove forest, and soft-bottom), SST and NPP in each cell. Statistical analyses were performed to test the relationship between the environmental variables versus four different species richness subgroups: combined all species, all fish species, all habitat-forming species (corals, mangroves, and seagrasses), and all invertebrate species. We modeled the influences of habitat area and heterogeneity to each subgroup using both a generalized linear model (GLM) and a spatial linear model (SLM) to account for spatial autocorrelation [78]. If autocorrelation is present, then a non-spatial GLM is not correct and will give biased estimates. We performed GLM using maximum likelihood estimation. Species richness is often considered as a form of count data, and therefore, the response variable *y* is fitted as a Poisson variable. Using the log of *y* we fit the GLM as below:
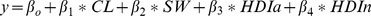
where *β_o_, β_1_*, *β_2_*, *β_3_*, and *β_4_* are the parameter estimates. For SLM, we used maximum likelihood estimation of spatial simultaneous autoregressive error model in the form:



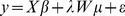
where *β* is the vector of coefficient for intercept and explanatory variable *X*; *λ* is the spatial autoregression coefficient; *W* is the spatial weights; *μ* is the spatially-dependent error term; and *ε* is the error vector. Likewise, the Poisson response variable *y* was also fitted as a log function.

We performed SLM with spatial distance weights derived from five nearest-neighbor cells. We used five and not eight nearest neighbors since most of the cells (especially in small grids) are isolated creating coastal and island effects. The presence of isolated cells will create a bias in spatial weight distance calculation. To normalize the data, all variables (dependent and independent) were log-transformed. Pearson correlation coefficients were generated for all between-variable comparisons. We tested individual habitat predictors versus each subgroup using single-predictor models. Then we used multiple-predictors model to test the effects of the combination of different habitats in each subgroup. The effects of multiple predictors were further explained by identifying the minimal-adequate model using backwards elimination method. Model fit was assessed using t-values (GLM) and z-values (SLM) for both single and multiple-predictor relationships. In GLM, adjusted *R^2^* values were used as a guide to model selection by evaluating the amount of variation explained. Since an equivalent *R^2^* does not exist in spatial analysis with a logistic regression, we used pseudo-*R^2^* [97] to evaluate the goodness-of-fit in SLM models. We calculated Akaike Information Criterion (AIC) to measure information content of models. Spatial autocorrelation was tested on model residuals using Moran’s *I* [98]. GLM analyses were performed using the open-source language R (R Core Development Team). SLM analyses were performed using the spdep package [99] in R statistical software.

## Results

### Species Richness

Species richness across the Indo-Pacific showed the expected pattern of highest richness in the Coral Triangle (Figure 1A). The central Philippines had the highest 2.5% of species richness, and the highest 10% was found in most of the Philippines and eastern Indonesia (Figure 1B). About 34% of all species in this study occurred in the north and central part of the Coral Triangle. The highest 30% of species richness radiated from this epicenter north to southern Japan, south to south-central Indonesia and the northeast tip of Australia, west to southern Sumatra and east to the easternmost Solomon Islands and species richness continued to diminish with distance from this epicenter (Figure 1A, 1B). Separate analysis for shore fishes, molluscs and crustaceans, and the habitat-forming primary producer species (corals, mangroves, and seagrasses) each showed a similar peak of species richness in the Coral Triangle (Figure 1C–E). The pattern of species richness of shore fishes (Figure 1C) was most similar to the combined analysis (Figure 1B), since fish species constituted the bulk of the species in the analysis, with a peak in the central Philippines. Molluscs and crustaceans appeared more concentrated in the northern part of the Coral Triangle (mostly around the Philippines) than shore fishes (Figure 1C, 1D). The highest 10% of species richness of corals, mangroves, and seagrasses was widely distributed throughout the Coral Triangle, with the highest 2.5% in the northern and central part (Figure 1E). The areas of least species richness in the combined analysis were in the eastern Pacific and along parts of the northern coasts of the Indian Ocean (Figure 1A). The low species richness in the open ocean represented the pelagic species found in coastal waters and was not representative of the total biodiversity of the open ocean.

### Habitat Availability and Heterogeneity

**Figure 2:**
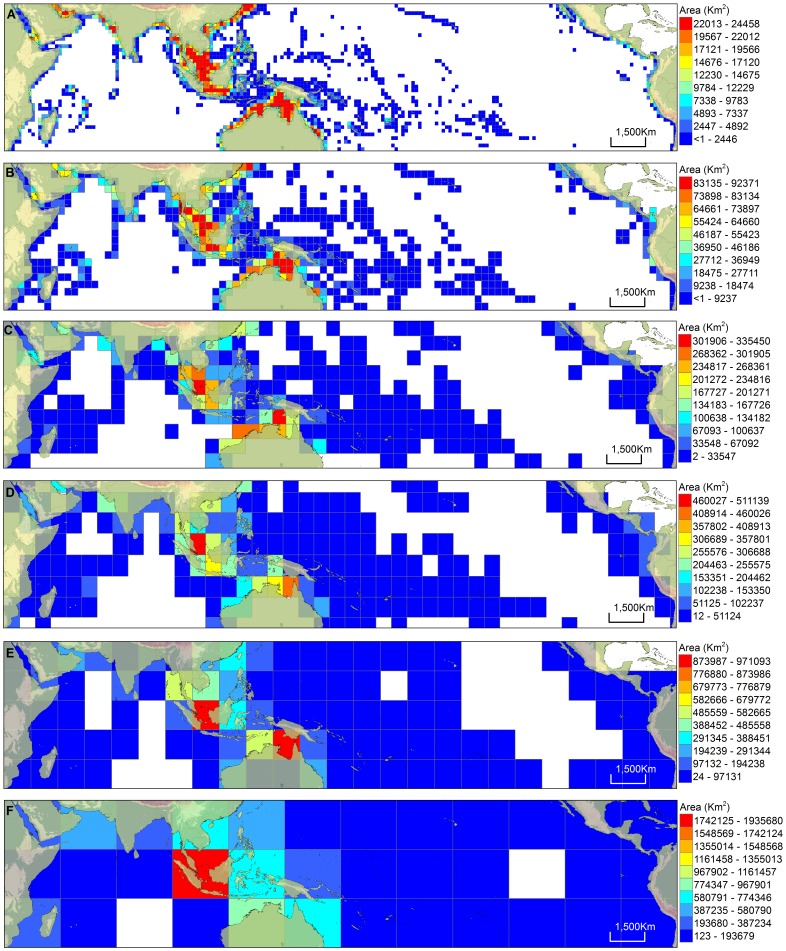
Distribution pattern of shallow water area extent in the Indo-Pacific at different grid scales. The grids were classified into 10 equal interval classes based on the amount of shallow water area recorded in each cell such that cells in red have the largest amount of shallow water area, and cells in blue have the lowest amount of shallow water area. Cells with zero values are not displayed. (A) Small grid, (B) Medium grid, (C) Large grid, (d) UTM grid, (E) Extra large grid, (F) Largest grid.

**Figure 3:**
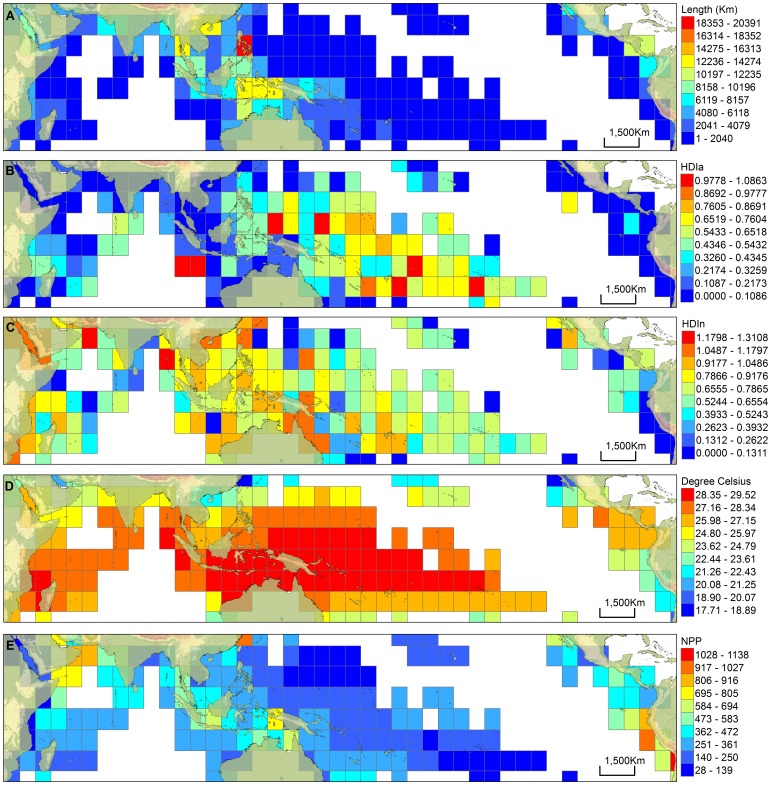
Distribution pattern of the different parameters in the Indo-Pacific using UTM grid. The grids were classified (equal interval) into 10 classes based on the amount of shallow water area recorded in each cell such that cells in red have the largest parameter value, and cells in blue have the lowest parameter value. Cells with zero values are not displayed. (A) Extent of coastline (km), (B) Habitat heterogeneity index using area (HDIa), (C) Habitat heterogeneity index using number (HDIn), (D) Sea surface temperature (SST), (E) Net primary productivity (NPP).

Greatest shallow water area (SW) and longest coastline (CL) per unit area (Figure 2A–F, 3A) were mostly concentrated in the Coral Triangle; however, these two habitat predictors were not evenly distributed within the Coral Triangle (Figures 2, 3A and Figure S9). As expected, the greatest concentration of shallow water area was over the Sunda and Arafura shelves, which are the two largest tropical shelf habitats in the world in terms of total area (Figure 2). These two large tropical shelf areas were reflected in all grid sizes (Figure 2A–E) except the largest grain size that shows a more diffuse concentration of shallow water around the Arafura Shelf (Figure 2F). The cells with the longest coastline mostly complemented rather than coincided with the large shallow water areas of the Sunda and Arafura shelves (Figures 2, 3A and Figure S9) in the Coral Triangle. The location of high coastline concentration generally was found in the Philippines and eastern Indonesia (Figures 3A and Figure S9), but with peaks outside the Coral Triangle at some grain sizes (Figure S9A–C). A peak in coastline length was located in the central Philippines at the UTM grain size (Figure 3A). The cells with the highest values of heterogeneity indices were prevalent in different locations dependent on the type of index used. The cells with high HDIa index values were concentrated mostly around oceanic island areas such as the central Pacific Ocean (Figure 3 and Figure S10). HDIn values were highest at widespread locations across the Indo-Pacific with generally high values found in the Coral Triangle and in areas north and east of the Coral Triangle that had the highest 30% of species richness in the Indo-Pacific (Figures 1B, 3 and Figure S11).

### SST and NPP

**Figure 4:**
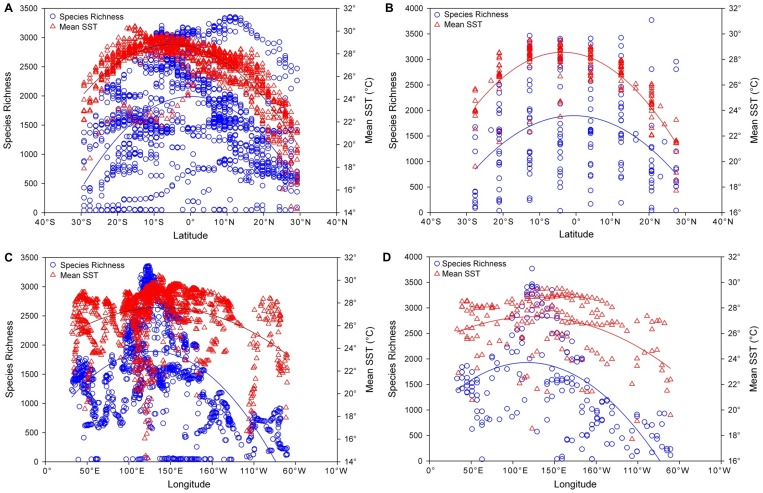
Species richness and mean SST versus latitude or longitude at different grid scales. (A) Latitude at small grid, (B) Latitude at UTM grid, (C) Longitude at small grid, (D) Longitude at UTM grid. Latitudinal peaks of species richness (blue circle) are shown along the 10–20° north and latitudinal peaks in mean SST values (°C) (red triangle) are along the 10–20° south. Longitudinal peaks of species richness (blue circle) are located in the 120° east while longitudinal peaks in mean SST values (°C) (red triangle) are found along the 130–150° east.

Average SST values were highest (over approximately 29°C) around and east of the Solomon Islands and northern Australia and were also high (over approximately 28°C) in eastern central Indonesia, western Sumatra and northwestern Madagascar (Figure 3D and Figure S12). Species richness correlated significantly with latitude (r = 0.440, p<0.001 for smallest grid size; r = 0.486, p<0.001 for UTM grid sizes) with peaks in species richness to the north of peaks in SST (Figure 4A, 4B). The longitudinal peak in SST of the Indo-Pacific Warm Pool corresponded closely with the eastern range of the peak in species richness (highest 10% to 30% of species richness; Figure 3B) and these two variables were significantly correlated longitudinally (r = 0.317, p<0.001 at smallest grid size; r = 0.377, p<0.001 at UTM grid size), although they did not closely co-vary elsewhere throughout their longitudinal range (Figure 4C, 4D). NPP values were highest in western South America, eastern China, between Australia and New Guinea, southern Borneo, and between Oman and India (Figure 3E and Figure S13).

### Predictors of Species Richness

Predictor variables were consistently highly correlated with one another at all scales, with some notable exceptions (Table 1). SW and CL had strong positive correlations with each other, with HDIn, and with NPP. SW had significant negative correlations with HDIa (except at the largest grid size) and CL was variably correlated with HDIa. SW and CL were insignificantly or negatively correlated with SST. NPP was most consistently negatively correlated with HDIa, insignificantly correlated with HDIn, and negatively or insignificantly correlated with SST except a moderate significant positive correlation at the medium grid size.

**Table 1:** Pearson correlation coefficients between all predictor variables within the same grid size.

	SW	CL	HDIa	HDIn	SST
**CL Small Grid**	0.382***	1			
**CL Medium Grid**	0.665***	1			
**CL Large Grid**	0.794***	1			
**CL UTM Grid**	0.794***	1			
**CL X Large Grid**	0.823***	1			
**CL Largest Grid**	0.909***	1			
**HDIa Small Grid**	−0.365***	0.164***	1		
**HDIa Medium Grid**	−0.241***	0.130**	1		
**HDIa Large Grid**	−0.426***	−0.135*	1		
**HDIa UTM Grid**	−0.357**	−0.102 ns	1		
**HDIa X Large Grid**	−0.392***	−0.081 ns	1		
**HDIa Largest Grid**	0.217 ns	0.218 ns	1		
**HDIn Small Grid**	0.046 ns	0.456***	0.488***	1	
**HDIn Medium Grid**	0.247***	0.445***	0.447***	1	
**HDIn Large Grid**	0.292***	0.366***	0.281***	1	
**HDIn UTM Grid**	0.377***	0.449***	0.301***	1	
**HDIn X Large Grid**	0.358***	0.399***	0.263*	1	
**HDIn Largest Grid**	0.578***	0.584***	0.457**	1	
**SST Small Grid**	−0.086**	−0.116***	0.129***	0.015 ns	1
**SST Medium Grid**	−0.033 ns	−0.026 ns	0.154***	0.096*	1
**SST Large Grid**	−0.087 ns	−0.060 ns	0.395***	0.234***	1
**SST UTM Grid**	0.005 ns	0.068 ns	0.464***	0.233**	1
**SST X Large Grid**	0.106 ns	0.221*	0.402***	0.357***	1
**SST Largest Grid**	0.279 ns	0.294 ns	0.246 ns	0.429*	1
**NPP Small Grid**	0.522***	0.107***	−0.426***	−0.121***	0.003 ns
**NPP Medium Grid**	0.511***	0.269***	−0.364***	0.011 ns	0.142**
**NPP Large Grid**	0.468***	0.337***	−0.415***	−0.033 ns	−0.211**
**NPP UTM Grid**	0.400***	0.254**	−0.437***	−0.075 ns	−0.257**
**NPP X Large Grid**	0.647***	0.545***	0.581***	0.001 ns	−0.213*
**NPP Largest Grid**	0.563**	0.625***	−0.109 ns	0.140 ns	−0.007 ns

The predictors are shallow water area (SW), coastline length (CL), habitat diversity based on area (HDIa), habitat diversity based on number of patches (HDIn), sea surface temperature (SST), and net primary productivity (NPP). Asterisks indicate significance value of P:

* (<0.05),

** (<0.01);

*** (<0.001); ns (not significant).

Grid sizes are as follows: Small = 23,000 km^2^; Medium = 92,000 km^2^; Large = 368,000 km^2^; UTM = 617,000 km^2^; Extra large = 1,470,000 km^2^; Largest = 5,100,000 km^2^.

Species richness of all taxonomic sets was significantly correlated with most environmental variables at all scales in single predictor GLM and SLM with the exception of insignificant correlations with SST at the smallest grid sizes, insignificant correlations with NPP at nearly all grid sizes, and HDIa and some other variables at the larger grain sizes (Table 2). CL most consistently explained the highest amount of variation with respect to species richness in both single predictor GLM and SLM except SW explained most variation at the smallest grain size, and HDIn was prominent at larger grain sizes for invertebrates and habitat-forming species (Table S4).

**Table 2:** Significant single-predictor Generalized Linear Model (GLM) and Spatial Linear Model (SLM) for species richness.

Subgroups	Grid Size	Single Predictor Generalized Linear Model						Single Predictor Spatial Linear Model					
		SW	CL	HDIa	HDIn	SST	NPP	SW	CL	HDIa	HDIn	SST	NPP
**All Species**	Small	***	***	***	***	ns	***	***	***	***	***	ns	*
	Medium	***	***	***	***	*	*	***	***	***	***	ns	ns
	Large	***	***	**	***	***	ns	***	***	***	***	***	ns
	UTM	***	***	**	***	***	ns	***	***	*	***	***	ns
	X Large	***	***	**	***	***	ns	***	***	ns	***	**	ns
	Largest	***	***	**	***	***	ns	***	***	ns	**	*	ns
**All Fish Species**	Small	***	***	***	***	ns	***	***	***	***	***	ns	*
	Medium	***	***	**	***	ns	**	***	***	**	***	ns	ns
	Large	***	***	ns	***	***	**	***	***	***	***	***	ns
	UTM	***	***	*	***	***	ns	***	***	**	***	***	ns
	X Large	***	***	ns	***	***	ns	***	***	ns	***	**	ns
	Largest	***	***	*	***	***	ns	***	***	ns	***	*	ns
**All invertebrates**	Small	***	***	***	***	ns	ns	***	***	***	***	ns	ns
	Medium	***	***	***	***	ns	ns	***	***	***	***	ns	ns
	Large	***	***	***	***	***	ns	***	***	***	***	**	ns
	UTM	***	***	***	***	***	ns	***	***	**	***	***	ns
	X Large	***	***	***	***	***	ns	*	**	*	***	*	ns
	Largest	***	*	*	***	***	ns	ns	ns	ns	**	***	ns
**All habitat-forming species**	Small	***	***	***	***	**	ns	***	***	***	***	ns	ns
	Medium	***	***	***	***	**	ns	***	***	**	***	ns	ns
	Large	***	***	***	***	***	ns	***	***	***	***	***	ns
	UTM	***	***	***	***	***	ns	***	***	**	***	***	ns
	X Large	***	***	***	***	***	ns	**	***	ns	**	***	ns
	Largest	**	*	*	***	***	ns	ns	ns	ns	*	***	ns

Actual *R* ^2^ and pseudo-*R* ^2^ values can be found in Table S4. The predictors are shallow water area (SW), coastline length (CL), habitat diversity based on area (HDIa), habitat diversity based on number of patches (HDIn), sea surface temperature (SST), and net primary productivity (NPP). Asterisks indicate significance value of P:

* (<0.05),

** (<0.01);

*** (<0.001); ns (not significant).

Grid sizes are as follows: Small = 23,000 km^2^; Medium = 92,000 km^2^; Large = 368,000 km^2^; UTM = 617,000 km^2^; Extra large = 1,470,000 km^2^; Largest = 5,100,000 km^2^. The significance value with the highest adjusted *R^2^* and pseudo-*R^2^* is highlighted in boldface.

Multiple regression results were similar to single predictor results in that many environmental variables were significant and retained in models (Table 3). GLM and SLM results for different taxonomic sets at different grain sizes were also similar in retaining specific environmental variables in models with the notable exception that HDIn, SST, and NPP variables mostly excluded from models at the two smallest grain sizes in the SLM. Moran’s *I* indicated significant autocorrelation at all grains sizes except the largest grain size. As expected with increasing grains size and decreasing sample sizes, *R* ^2^ and pseudo-*R* ^2^ increased (less variation to explain) and AIC values decreased with larger grain sizes (less entropy to account for). However, when comparing equal grain sizes between GLM and SLM the correction for significant autocorrelation always improved the model (higher pseudo-*R* ^2^ versus *R* ^2^ and lower AIC). CL was consistently retained in all SLM for all taxonomic sets at all grain sizes except in the largest grain size. In SLM across all taxa most consistently retained were SW at smallest and largest grain sizes, HDIa at smallest grain size and variably in large grain sizes, HDIn and SST mostly at all grain sizes except small and medium, and NPP negatively correlated and variably retained at larger grain sizes.

**Table 3:** Minimal adequate multiple-predictor Generalized Linear Model (GLM) and Spatial Linear Model (SLM) results for all grid sizes.

Models	Grid Size	SW	CL	HDIa	HDIn	SST	NPP	Moran’s *I*	*R^2^*/p-*R^2^*	AIC
**GLM (t-values) for all species**	Small	11.138***	7.520***	4.888***	2.570*	2.262*		0.4468***	0.236	3719.64
	Medium	4.551***	8.167***		3.552***	3.281**	−3.654***	0.2847***	0.369	1354.75
	Large	3.416***	4.509***	3.667***	3.160**	5.077***		0.1914***	0.573	411.74
	UTM		10.087***		3.456***	7.440***		0.2314***	0.604	328.53
	X Large		10.089***		6.240***	4.686***	−3.552***	0.1888***	0.804	100.27
	Largest	6.864***			2.918**	4.005***	−4.084***	−0.0443 ns	0.871	**23.17**
**SLM (z-values) for all species**	Small	8.192***	4.267***	2.839**				−0.0110	0.508	**3109.00**
	Medium	3.643***	6.932***					−0.0140	0.486	**1238.30**
	Large		10.873***	2.490*	2.218*	4.885***		−0.0214	0.618	**392.04**
	UTM		9.885***		2.346*	5.421***		−0.0161	0.656	**308.67**
	X Large		10.640***		4.567***	3.282**	−2.243*	−0.0119	0.837	**88.74**
	Largest	9.699***			3.642***	3.727***	−7.754***	0.0383	0.878	23.22
**GLM (t-values) for all fish species**	Small	11.621***	8.375***	4.869***	2.481*			0.4488***	0.257	3384.89
	Medium	5.291***	8.708***		3.380***	3.027**	−3.220**	0.2740***	0.407	1209.05
	Large	3.503***	5.351***	2.712**	3.304**	5.009***		0.2085***	0.606	348.05
	UTM		12.679***		3.411***	7.009***		0.1890***	0.664	275.83
	X Large	3.279**	7.003***		5.858***	4.960***	−3.572***	0.1096*	0.847	51.58
	Largest	7.520***			2.863**	3.318**	−3.647**	−0.1213 ns	0.859	19.12
**SLM (z-values) for all fish species**	Small	8.817***	4.228***	2.649**				−0.0072	0.529	**2756.30**
	Medium	4.539***	6.860***					−0.0159	0.510	**1101.30**
	Large	2.023*	5.439***	2.363*	2.310*	4.868***		−0.0121	0.657	**324.40**
	UTM		11.934***		2.585**	5.466***		−0.0110	0.696	**262.84**
	X Large		11.697***		4.915***	3.769***		−0.0150	0.861	**45.58**
	Largest	12.855***			3.973***	2.558*	−9.571***	0.0246	0.901	**13.35**
**GLM (t-values) for invertebrates**	Small	8.797***	6.980***	5.662***		2.093*	−2.028*	0.4593***	0.160	4935.93
	Medium		10.655***		2.768**	1.990*	−3.136**	0.2608***	0.250	2011.48
	Large	7.800***		4.381***	3.191**	2.786**		0.2284***	0.414	690.23
	UTM		4.746***		3.155**	6.161***		0.2481***	0.390	522.40
	X Large		5.304***	1.995*	5.186***	2.574*	−2.498*	0.3689***	0.657	227.71
	Largest	3.060**			3.270**	3.391**	−4.541***	0.2722**	0.743	60.89
**SLM (z-values) for invertebrates**	Small	6.527***	4.116***	2.825**				−0.0153	0.450	**4347.70**
	Medium		10.755***					−0.0166	0.358	**1922.20**
	Large		9.430***	4.765***		2.787**		−0.0249	0.485	**665.64**
	UTM		7.003***			4.218***		−0.0242	0.480	**497.54**
	X Large	−3.918***	8.575***		3.910***			−0.0310	0.814	**172.55**
	Largest				3.110**	3.883***		−0.0008	0.885	**35.79**
**GLM (t-values) for habitat-forming species**	Small	11.103***	4.753***	4.130***	4.727***	4.027***	−3.908***	0.5059***	0.219	5342.45
	Medium	4.682***	6.195***		4.724***	4.316***	−5.316***	0.2778***	0.328	2121.49
	Large	3.206**	2.475*	4.325***	3.565***	5.298***		0.1602***	0.505	762.75
	UTM	6.337***		1.988*	3.925***	7.047***		0.2102***	0.562	548.46
	X Large		5.071***		5.419***	5.113***	−2.889**	0.3229***	0.677	264.06
	Largest				4.476***	5.266***		0.0716 ns	0.711	**81.14**
**SLM (z-values) for habitat-forming species**	Small	7.648***	3.840***	2.807**				−0.0203	0.559	**4548.50**
	Medium	3.552***	5.311***		2.015*		−2.026*	−0.0100	0.456	**2006.30**
	Large	9.606***	4.202***			4.367***		−0.0220	0.545	**746.99**
	UTM		6.710***		3.300***	4.983***		−0.0236	0.628	**524.21**
	X Large		5.761***		2.663**	3.724***	−2.517*	−0.0211	0.787	**230.56**
	Largest	2.496*			3.510***	5.445***	−2.800**	0.0040	0.770	81.54

The predictors are shallow water area (SW), coastline length (CL), habitat diversity based on area (HDIa), habitat diversity based on number of patches (HDIn), sea surface temperature (SST), and net primary productivity (NPP). Values under predictor variables are t-values for GLM and z-values for SLM. Asterisks indicate significance value of P:

* (<0.05),

** (<0.01);

*** (<0.001); ns (not significant). Grid sizes are as follows: Small = 23,000 km^2^; Medium = 92,000 km^2^; Large = 368,000 km^2^; UTM = 617,000 km^2^; Extra large = 1,470,000 km^2^; Largest = 5,100,000 km^2^. *R^2^* values are for GLM results, while pseudo-*R^2^* (p-*R^2^*) values are for SLM results. The lower AIC values between SLM and GLM of the same grid size per group are highlighted in boldface. Lower AIC values suggest that SLM models are preferred in most cases than its GLM counterparts.

## Discussion and Conclusions

Range overlap (Figure 1A) of 10,446 expert-based generalized distributions of fishes, invertebrates (molluscs and crustaceans), and habitat-forming species (corals, seagrasses, and mangroves) recovered the classic pattern of decreasing species richness with distance from the Coral Triangle [1]. The highest concentration of species richness within the Coral Triangle, i.e., in the Philippines and eastern Indonesia, was similar to what has been proposed by Veron et al. (2009) [3] based on corals. However, the peak in biodiversity in this region comprises more than corals and coral reefs. Other important habitat-forming biota such as seagrasses and mangroves peak in species richness in this region (Figure 1E) and also substantially support many species [100]–[101] and form complex ecosystems in conjunction with coral reefs [102]. Therefore, in addition to the Philippines, eastern Sabah, eastern Indonesia, Timor L’Este, New Guinea, and Solomon Island delineation [3], we recommend that western Indonesia, western Sabah, Brunei, Singapore, and peninsular Malaysia should also be considered as part of the Coral Triangle (red and pink areas in Figure 1E).

The pattern of species richness in this study (Figure 1A) is dominated by the preponderance of shore fishes, so generalizations from our results are strongest for shore fishes because the data are nearly complete for this group. However, the pattern of species richness in the more limited subset of macroinvertebrates (Figure 1D) is consistent with what is observed in shore fishes (Figure 1C). The peak in diversity of invertebrates is more concentrated in the northern apex of the Coral Triangle than shore fishes. A part of this may be due to a higher density of sampling effort for invertebrates in the compared to elsewhere in the Coral Triangle [74]. The tighter pattern of peak range overlap may also be related to the differences in mobility between the two groups. Most fishes and invertebrates have pelagic larval stages with durations that may or may not be related to range size [103] but fishes are typically more mobile as adults than macroinvertebrates, which include many fixed or slow-moving benthic species as adults. The nearly complete set of expert-based range maps for the coastal fishes, the corroboration of peaks in species richness among different taxonomic sets (Figure 1), and the radiating pattern from the epicenter (Figure 1B) strongly supports an epicenter of species richness within the Coral Triangle (Figure 1A). The often-posed question of what factors contribute to the epicenter of marine species richness of the Coral Triangle, therefore, can be focused toward identification of predictors of species richness in the northern apex and central region of the Coral Triangle.

The high correlation among our predictor variables (Table 1) and the very high frequency of single predictor significant models (Table 2) makes it challenging to identify any one predictor of species richness. Fortunately, NPP is significant in single predictor models, but is infrequently retained in multiple predictor models (Table 3) and when retained, mostly negative. NPP is highly correlated with SW and CL because of elevated productivity in shallow sunlit coastal areas. NPP is highly negatively correlated with HDIa because habitat complexity is high around oceanic islands where NPP is low. SST and NPP are not highly correlated as the Indo-Pacific Warm Pool is largely oligotrophic [30]. The reduced importance of NPP as a factor in species richness is similar to what was observed on a global scale [57]. The negatively significant retention of NPP in multiple predictor models may be because higher NPP is typically associated with more turbid coastal waters or cooler upwelling waters, both of which are inconsistent with nutrient limited, highly diverse coral reef ecosystems. Therefore, the idea that more available energy promotes more species because of reduced partitioning of available food resources [48] is not supported in our models. SST also gives a clear signal in that it is insignificantly or negatively correlated with SW and CL and significantly correlated with HDIa also because of the oceanic coverage of the Indo-Pacific Warm Pool. However, the retention of SST and the habitat availability predictors in multiple-predictor models (Table 3) is variable and often depends on scale and autocorrelation.

The choice of best predictors for species richness (Table 3) is influenced by resolution, scale, and autocorrelation. The correction for autocorrelation and for that matter, the use of regression models in spatial analyses of species richness is somewhat controversial [104], [105]. Moran’s *I* test shows significant effects of autocorrelation in our data (Table 3) similar to what was observed by Bellwood et al. (2005) [53] and Tittensor et al. (2010) [57]. The rank of predictor variables without correction for autocorrelation was similar to the rank with correction except that fewer predictors were retained in SLM. However, it is clear that correction of autocorrelation in SLM (Table 3) consistently resulted in more variation explained (higher pseudo-*R* ^2^ than *R* ^2^) and more entropy removed from the model (lower AIC). When comparing models based on the same grid size, correction for autocorrelation resulted in a lower AIC, and therefore, SLM is the preferred model [106], [107] in all cases for all species groups except for the largest grain where tests for autocorrelations were insignificant or not highly significant (Table 3). Therefore, the SLM model is preferred in all cases except the largest grid size for all species and habitat-forming species taxonomic subsets, where GLM is preferred.

The choice of an optimal grid or grain size is not straightforward because model choice using different data sets (e.g., with different sample sizes) is not statistically defensible [106], cf. [57]. As expected, *R* ^2^ values increase with increasing grid size over the same area since there is less variation to explain with smaller sample sizes (Table 3). Similarly, AIC values decrease with larger grid sizes because there is less entropy to account for in smaller sample sizes. In fact, there may not be an optimal grain size in analyses that attempt to explain spatial variation in species richness [79]. The choice of grid size will ultimately depend on practical and ecological considerations such as the presence or absence of a predictor variable at different grain sizes. For example, a value for coastline length may not be found in a grid over a continental shelf at small grain sizes. Therefore, optimal grid size will mostly depend on the predictor variables chosen to explain variation in species richness.

Habitat availability predictor variables (SW, CL, HDIa, HDIn) and the available kinetic energy predictor variable (SST) are consistently retained as positive significant predicator variables across species groups and grid sizes, accounting for autocorrelation (Table 3). Coastline length (CL) is most consistently retained for all grid sizes except the largest grid size and CL most consistently explained the highest amount of variation in species richness in single predictor spatial models (Table 2). Our identification of CL as a powerful proxy for habitat availability is consistent with the results of Etnoyer (2001) [36] and Tittensor et al. (2010) [57]. However, CL does not capture all aspects of habitat availability (or some other factor) because at least one other habitat availability variable is retained in SLM at all grid sizes that CL is retained. This suggests that both habitat area and habitat complexity components may be needed to best test the ‘area of refuge’ hypothesis [32] for the Coral Triangle epicenter of species richness. Our results may help to guide selection of habitat availability variables that can predict variation in marine species richness at different grid sizes, since different habitat predictors operate at different spatial grains [79]. For example, in the shore fishes, shallow water area and heterogeneity (HDIa) should be considered at grain sizes equivalent to our small and medium grid sizes. At the large equivalent grain size all habitat availability variables should be considered. For UTM and grain sizes around 5 million km^2^ a habitat heterogeneity index similar to HDIn may suffice in addition to CL as a proxy for habitat availability. Differences in how the different habitat complexity indices behave in models are a result of the form of complexity they emphasize. Our HDIa index is based on the relative proportion of area for our different habitat types (soft bottom, coral reef, seagrass, and mangrove) in a grid. Heterogeneity is negatively correlated with shallow water area (Table 1) because soft sediment habitats will have proportionally larger area in grids with extensive continental shelf area. This keystone habitat type will dominate (reducing diversity of habitats) in these grids while oceanic island grids without extensive shelf area will score highly (Figure 3D). HDIn is calculated based on the number of polygons of the different habitat types found in each grid, and therefore, dominance of any one habitat type is minimized. This habitat complexity index is more concentrated in coastal areas and it consistently explains variation in species richness at larger grid sizes when proximity to other coastal areas is reduced by autocorrelation correction (Table 3). Further development of these types of habitat complexity indices may be possible as GIS layers that map the different nearshore habitats become more available and more refined. More refined GIS layers may also help address habitat complexity in terms of habitat or environmental gradients that may also influence species richness patterns [68], [69], [108]. Heterogeneity indices like these may help more clearly identify keystone structures as indicators of species richness [64]–[66].

The position of the Indo-Pacific Warm Pool appears to be a primary reason that available kinetic energy, as exemplified by SST, is most consistently retained in models with autocorrelation considered (Table 3). Latitudinal variations in SST will account for some variation in species richness although the epicenter of species richness at the apex of the Coral Triangle (Figure 1) occurs north of peaks in SST (Figure 4A, 4B). This epicenter of species richness corresponds more closely with peaks in CL (Figure 3A) than SST (Figure 3D), and CL is consistently and strongly retained in models (Table 3). The longitudinal peaks in SST correlate significantly with species richness. They spatially corresponded with the secondary 10–30% area of species richness (Figures 3B, 4C, 4D). The primary longitudinal peak in species richness is best explained by the area of highest concentration of CL while the secondary 10–30% area of species richness is best explained by the longitudinal peak in SST corresponding to the Indo-Pacific Warm Pool. Bellwood et al. (2005) [53] dismissed the importance of SST in their analysis of Indo-Pacific species richness patterns of fishes in favor of a mid-domain effect. Their peak in species richness from a limited subset of fishes was far to the east of the epicenter shown from the more complete data set we introduce here (Figure 1). Their epicenter corresponded a little more closely with their choice of the mid-domain which also corresponded with the center of the Indo-Pacific Warm Pool. In contrast, the main conclusion of Tittensor et al. (2010) [57] was that SST is the primary factor in shaping global marine biodiversity (having dismissed the choice of a mid-domain as indefensible) and that available kinetic energy promotes higher rates of speciation in the tropics. However, their conclusion was based on strong latitudinal effects reinforced by long-held observations that fewer species are found at higher latitudes. Tittensor et al. (2010) [57] did not specifically address the fact that the peak in species richness of the bulk of their dataset, the tropical coastal fishes, may have coincided with the position of the Indo-Pacific Warm Pool. The position of the Indo-Pacific Warm Pool correlates with a portion of the peak in species richness in our data set and this also could have been a factor in the dataset of Tittensor et al. (2010) [57].

Our results suggest that kinetic energy, habitat availability, and habitat complexity are all important in shaping species richness patterns across the Indo-Pacific but the relationship of these variables to area of refuge, area of accumulation, and center of origin hypotheses is not straightforward. The striking pattern of diminishing species richness away from the epicenter in the Coral Triangle was a factor in formation of both the center of origin and area of accumulation hypotheses, both mediated primarily by dispersal out of or into the Coral Triangle [1]. The center of origin hypothesis relies on speciation within the Coral Triangle and dispersal away from the speciation center while the area of accumulation hypothesis relies on speciation outside the Coral Triangle and dispersal into the Coral Triangle. The latter hypothesis relies on the observation that prevailing currents that could dominate dispersal are mostly toward the Coral Triangle from Oceania where numerous isolated islands could promote allopatric speciation [45], [46]. The position of the Indo-Pacific Warm Pool over a great many of these isolated islands outside the Coral Triangle could also promote speciation as warmer temperatures are thought to hasten population turnover times and, therefore, hasten rates of evolution [70]. There is some evidence for speciation on these peripheral islands [109] but this is only one example out of the highly diverse Indo-Pacific biota. However, if the Indo-Pacific Warm Pool does hasten speciation and extinction over these numerous isolated islands, the pattern of increasing species richness into the Coral Triangle from the east (Figure 1B) corresponds closely to this hypothesis. Ample available habitat may provide refuge for peripherally-derived species and the cooler temperature within the Coral Triangle may also depress population turnover times and therefore, rates of extinction.

An alternative explanation for the pattern of increasing species richness into the Coral Triangle from the east and the correlation of SST with species richness is that the position of the Indo-Pacific Warm Pool is only a coincidence and does not influence speciation and extinction rates. If speciation within the Coral Triangle is the dominant factor then dispersal out of the Coral Triangle would rely on stepping stones of available habitat counter to prevailing currents over geologic time periods. There is some evidence for gene direction out of the Coral Triangle [110] and a preponderance of Indo-Pacific species originating from the Miocene [42], so there would be sufficient geological time to disperse out of the Coral Triangle against prevailing currents. Teasing apart whether area of accumulation or center of origin is most important may hinge on our ability to demonstrate whether diversification of a lineage is most common within the Coral Triangle [4] or outside the Coral Triangle within the Indo-Pacific Warm Pool. Both of these hypotheses rely on the existence of available habitat to serve as refuge or as a means to promote speciation.

Habitat availability is central to both the center of origin and area of accumulation hypotheses but also to the neutral and eclectic hypotheses as well. The observed correlations among species richness, habitat availability, and habitat heterogeneity may simply be a function of the well-known species-area relationship [32]. This relationship could be interpreted as a neutral, probabilistic process wherein greater area provides higher probability of finding more species, or, more area provides a greater variety of habitats that provide more niches for species to fill [1]. Coastline length is a strong predictor for species richness in models and should be considered as a proxy for greater area or more available niches. Alternatively or additionally, more habitat may provide more refuge from extinction [32], [63] which is the same as enhanced survival in eclectic hypotheses that prefer multiple hypotheses as an explanation for the epicenter of diversity in the Coral Triangle [6], [8], [9], [20], [35], [42], [49]–[51]. To complicate these hypotheses further, habitat complexity may alternatively or additionally provide favorable conditions for either sympatric or allopatric speciation [111]. The presence of extensive and complex habitat potentially creates barriers to gene flow promoting speciation at both close range and wide range spatial scales. Both forms of speciation could be mediated by fluctuations in sea level [111], [112] and dramatic changes in ocean circulation that took place during the geological formation of the Coral Triangle [26].

This complex view of the area of refuge hypothesis supports the concept that both area of available habitat and complexity of available habitat are potentially important in models seeking to predict and conserve tropical marine species diversity. Shallow water area, as a measure of available habitat, is a significant component in shaping species richness in the region across different taxonomic groups. However, the largest shallow water area (the Sunda and Arafura shelves), does not coincide with the location of highest species richness (the Philippines, eastern Sabah, and eastern Indonesia). Available habitat in terms of shallow water area alone is not the optimal explanation for variation in species richness because coastline length and habitat heterogeneity are significant explanations for variation in species richness (Table 3). Bellwood and Hughes (2001) [10] used shallow water area alone to represent area of refuge and found that it explained a significant amount of variation in species richness. Their observed conclusions may have been stronger if they had also included a component of habitat complexity such as coastline length and a habitat diversity index. Spatial distribution and statistical analyses both show that coastline length is a better predictor for species richness than shallow water area. The longest coastline per unit area is most consistently found in the Philippines and eastern Indonesia at all grids (Figure S9) – the same locations where the highest 10% of species richness is found (Figure 1). The amount of variation in species richness explained by coastline concentration is consistently higher than that explained by shallow water area.

Another factor that may profoundly influence the role that available shallow water area plays in shaping biodiversity in the Coral Triangle is local extinction from glacial maxima and concomitant sea level lows. Marine life was extirpated on the extensive tropical sea floor represented by the Sunda and Arafura shelves several times during the Pleistocene [1]. A comparison of Figures 1B and 2A shows a surprising complementarity in the highest 30% range overlap of species richness with these extensive shelf areas that suggests that there may still be a limitation to marine species richness remaining from recent ice ages. This is a hypothesis that warrants further testing and could have implications for our understanding of marine connectivity beyond calibrating molecular clocks [113].

Tittensor et al. (2010) [57] showed that coastline length, as originally demonstrated by Etnoyer (2001) [36], is significant in explaining variation in species richness. Their conclusions may also have been strengthened by inclusion of an index of available area and habitat complexity and the use of expert-derived range maps [86]. The Coral Triangle has the most concentrated coastline (km of coastline per grid) in the Indo-Pacific region, contributed by the numerous islands in the archipelagos of the Philippines and Indonesia. Although the availability of shallow water habitat is important, a more complex shoreline is also important for many fishes and invertebrates during their early life history stages [114]. The complexity of shoreline in the Coral Triangle also resulted in an increase in habitat complexity, along with the concurrent increase in coral-carbonate platforms that contributes to species diversification [25].

Our findings further support previous studies based largely on different distribution databases [9], [67], [74], [115] that indicate that the global peak in species richness of shallow nearshore marine biota is in the central Philippines between southern Luzon and northern Mindanao (Figure 1). This is in spite of a more intense sampling of shore fishes that make up the bulk of our data in Indonesia than the Philippines both recently [51] and historically. Indonesia is listed as the type locality for more marine fishes than any other country because of intense periods of collections in Indonesia by Pieter Bleeker and earlier ichthyologists [116], [117] while the Philippines has relatively meager colonial natural history collections prior to the 20^th^ century [118]. In addition to habitat availability correlates with this species richness peak in the Philippines and the potential for cooler temperatures in the northern Coral Triangle moderating extinction rates, the temperature-latitudinal range in this area may also contribute to species richness. This region includes warm temperate species (e.g. [119]) that are not likely to be found in the more equatorial portion of the Coral Triangle. The Philippines also has more extensive shallow soft bottom shelf area than eastern Indonesia where species richness is marginally lower than the Philippines. This additional available habitat in the Philippines would allow the addition of many soft bottom species that will not be present in eastern Indonesia. In contrast to the finding in this study that shows a peak in shore fish species richness in the Visayan region of the Philippines (Figure 1C), one study indicates that eastern Indonesia may eventually overtake the central Philippines in terms of species richness of coral reef fishes [51]. However, Allen’s (2007) [67] comprehensive analysis supported higher species richness in the Philippines and Allen and Adrim (2003) [51] based their prediction on new species recently described and a very high level of sampling in eastern Indonesia, compared to the Philippines. It is likely that new species discovered in eastern Indonesia will eventually be found in the Philippines, particularly if a similar level of sampling effort were to take place in the Philippines. However, it should be emphasized that the peak in species richness delineated in this study is based on historical distribution records and there is evidence of exploitation-related biodiversity loss in the central Philippines [115]. Clearly, the central Philippines needs to redouble efforts in marine conservation to recover from and prevent further biodiversity loss in this unique global marine biodiversity epicenter. However, the pristine nature of many areas of the Bird’s Head region of Indonesia should also be the focus of intense marine conservation efforts to preserve the rich diversity there.

Available habitat and habitat complexity both significantly explain variation in species richness, and therefore, the loss of these attributes may reduce diversity. Advances in our understanding of the different components of area of refuge may help more clearly define distinctions between available habitat and complexity of habitat. For example, more accurate mapping of presumptive keystone structures such as seagrass beds, mangrove areas, and coral reefs may improve the accuracy of habitat diversity indices. Testing additional types of habitat indices may also improve our understanding. Finding a practical alternative to grid-based spatial analyses may also improve resolution of the degree to which habitat availability components and factors such as SST explain variation in species richness. It is clear that habitat-based conservation efforts targeted at preserving biodiversity should aim to preserve both area and complexity of habitats. This effort is most crucial in the Coral Triangle where species richness is at its peak but it may also be very important in preserving the evolutionary potential of isolated islands, particularly those in the Indo-Pacific Warm Pool. Habitat destruction is a key factor linked to species threats in the Coral Triangle but fortunately, destruction is less intense at present in the islands of Oceania [2]. Continued habitat loss and unrestrained exploitation [115] has the potential for a maximal threat in the Coral Triangle epicenter of species richness.

## Supporting Information

Figure S1 **Distribution pattern of shallow water extent in the Indo-Pacific at UTM grids shifted into different orientations.** The grids were classified (equal interval) into 10 classes based on the amount of shallow water area recorded in each cell such that cells in red have the largest amount of shallow water area, and cells in blue have the lowest amount of shallow water area. (A) UTM shifted north/south, (B) UTM shifted east/west, (C) UTM shifted northeast/southwest.
(PDF)

Figure S2 **Distribution pattern of coastal length extent in the Indo-Pacific at UTM grids shifted into different orientations.** The grids were classified (equal interval) into 10 classes based on the amount of coastal length recorded in each cell such that cells in red have the largest amount of coastal length, and cells in blue have the lowest amount of coastal length. (A) UTM shifted north/south, (B) UTM shifted east/west, (C) UTM shifted northeast/southwest.
(PDF)

Figure S3 **Distribution pattern of the habitat heterogeneity index using area in the Indo-Pacific at UTM grids shifted into different orientations.** The grids were classified (equal interval) into 10 classes based on the index values recorded in each cell such that cells in red have the largest index values, and cells in blue have the lowest index values. (A) UTM shifted north/south, (B) UTM shifted east/west, (C) UTM shifted northeast/southwest.
(PDF)

Figure S4 **Distribution pattern of the habitat heterogeneity index using number in the Indo-Pacific at UTM grids shifted into different orientations.** The grids were classified (equal interval) into 10 classes based on the index values recorded in each cell such that cells in red have the largest index values, and cells in blue have the lowest index values. (A) UTM shifted north/south, (B) UTM shifted east/west, (C) UTM shifted northeast/southwest.
(PDF)

Figure S5 **Distribution pattern of the mean sea surface temperature in the Indo-Pacific at UTM grids shifted into different orientations.** The grids were classified (equal interval) into 10 classes based on the index values recorded in each cell such that cells in red have the highest temperature, and cells in blue have the lowest temperature. (A) UTM shifted north/south, (B) UTM shifted east/west, (C) UTM shifted northeast/southwest.
(PDF)

Figure S6 **Distribution pattern of the mean net primary productivity in the Indo-Pacific at UTM grids shifted into different orientations.** The grids were classified (equal interval) into 10 classes based on the index values recorded in each cell such that cells in red have the highest productivity, and cells in blue have the lowest productivity. (A) UTM shifted north/south, (B) UTM shifted east/west, (C) UTM shifted northeast/southwest.
(PDF)

Figure S7 **Combined adjacent cells (red color) against the regular sized cells (yellow color).** (A) Small grid, (B) Medium grid, (C) Large grid, (D) UTM, (E) UTM shifted north/south, (F) UTM shifted east/west, (G) UTM shifted northeast/southwest. There are no adjacent cells that were combined in extra large and largest grids.
(PDF)

Figure S8 **The two types of basemaps used in this study.** (A) Visualization basemap consisting of 200 m depth and 100 km buffer, (B) Bathymetry basemap showing the 200 m depth used in analyses of species richness versus independent variables.
(PDF)

Figure S9 **Distribution pattern of coastal length extent in the Indo-Pacific at different grid scales.** The grids were classified (equal interval) into 10 classes based on the amount of coastal length recorded in each cell such that cells in red have the largest amount of coastal length, and cells in blue have the lowest amount of coastal length. (A) Small grid, (B) Medium grid, (C) Large grid, (D) Extra large grid, (E) Largest grid.
(PDF)

Figure S10 **Distribution pattern of the habitat heterogeneity index using area in the Indo-Pacific at different grid scales.** The grids were classified (equal interval) into 10 classes based on the index values recorded in each cell such that cells in red have the largest index values, and cells in blue have the lowest index values. (A) Small grid, (B) Medium grid, (C) Large grid, (D) Extra large grid, (E) Largest grid.
(PDF)

Figure S11 **Distribution pattern of the habitat heterogeneity index using number in the Indo-Pacific at different grid scales.** The grids were classified (equal interval) into 10 classes based on the index values recorded in each cell such that cells in red have the largest index values, and cells in blue have the lowest index values. (A) Small grid, (B) Medium grid, (C) Large grid, (D) Extra large grid, (E) Largest grid.
(PDF)

Figure S12 **Distribution pattern of the mean sea surface temperature in the Indo-Pacific at different grid scales.** The grids were classified (equal interval) into 10 classes based on the index values recorded in each cell such that cells in red have the highest temperature, and cells in blue have the lowest temperature. (A) Small grid, (B) Medium grid, (C) Large grid, (D) Extra large grid, (E) Largest grid.
(PDF)

Figure S13 **Distribution pattern of the mean net primary productivity in the Indo-Pacific at different grid scales.** The grids were classified (equal interval) into 10 classes based on the index values recorded in each cell such that cells in red have the highest productivity, and cells in blue have the lowest productivity. (A) Small grid, (B) Medium grid, (C) Large grid, (D) Extra large grid, (E) Largest grid.
(PDF)

Table S1 **Single predictor Generalized Linear Model (GLM) and Spatial Linear Model (SLM) results at different UTM grid orientations.** The predictors are shallow water area (SW), coastline length (CL), habitat diversity based on area (HDIa), habitat diversity based on number of patches (HDIn), sea surface temperature (SST), and net primary productivity (NPP). Values under predictor variables are t-values for GLM and z-values for SLM. Asterisks indicate significance value of P: *(<0.05), **(<0.01); ***(<0.001); ns (not significant). UTM NS = centroid shifted on a North-South plane, UTM EW = centroid shifted on an East-West plane and UTM NE = centroid shifted along a Northeast-Southwest plane. Sample size for each grid (n) is shown. The highest adjusted *R^2^* (GLM) and pseudo-*R^2^* (p–*R^2^*; SLM) value within each grid size are highlighted in boldface.
(PDF)

Table S2 **Multiple predictor Generalized Linear Model (GLM) and Spatial Linear Model (SLM) results at different UTM grid orientations.** The predictors are shallow water area (SW), coastline length (CL), habitat diversity based on area (HDIa), habitat diversity based on number of patches (HDIn), sea surface temperature (SST), and net primary productivity (NPP). Values under predictor variables are t-values for GLM and z-values for SLM. Asterisks indicate significance value of P: *(<0.05), **(<0.01); ***(<0.001). UTM NS = centroid shifted on a North-South plane, UTM EW = centroid shifted on an East-West plane and UTM NE = centroid shifted along a Northeast-Southwest plane. Sample size for each grid (n) is shown. *R^2^* values are for GLM results, while pseudo-*R^2^* (p–*R^2^*) values are for SLM results.
(PDF)

Table S3 **List of families with the number of species distribution maps used in this study.**
(PDF)

Table S4 **Single predictor Generalized Linear Model (GLM) and Spatial Linear Model (SLM) complete results at different grid sizes.** The predictors are shallow water area (SW), coastline length (CL), habitat diversity based on area (HDIa), habitat diversity based on number of patches (HDIn), sea surface temperature (SST), and net primary productivity (NPP). Values under predictor variables are t-values for GLM and z-values for SLM. Asterisks indicate significance value of P: *(<0.05), **(<0.01); ***(<0.001); ns (not significant). Sample size for each grid (n) is shown. The highest adjusted *R^2^* (GLM) and pseudo-*R^2^* (p–*R^2^*; SLM) value within each grid size are highlighted in boldface.
(PDF)

Text S1 **Methods and results for UTM grids shifted to different centroid orientation.**
(PDF)

Text S2 **Sources for species range used in producing the distribution maps.**
(PDF)
