# Supplementary material for: Habitat Availability and Heterogeneity and the Indo-Pacific Warm Pool as Predictors of Marine Species Richness in the Tropical Indo-Pacific
Source: PLoS One. 2013 Feb 15;8(2):e56245. doi: 10.1371/journal.pone.0056245 (PMC3574161; doi:10.1371/journal.pone.0056245)

Figure S1 Distribution pattern of shallow water extent in the Indo-Pacific at UTM grids shifted into different orientations.

The grids were classified (equal interval) into 10 classes based on the amount of shallow water area recorded in each cell such that cells in red have the largest amount of shallow water area, and cells in blue have the lowest amount of shallow water area. (A) UTM shifted north/south, (B) UTM shifted east/west, (C) UTM shifted northeast/southwest.

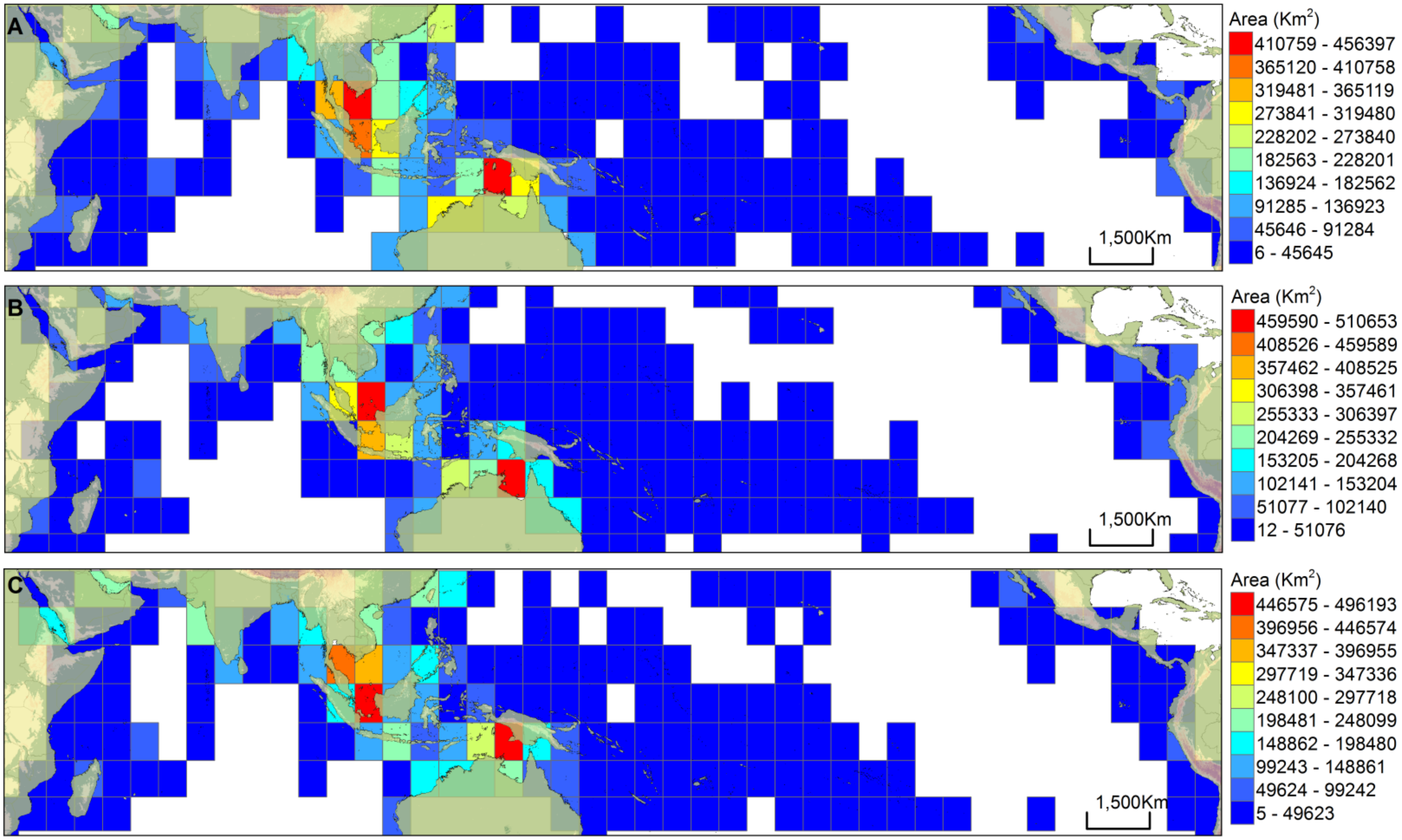

Supplement: Figure S1 — Distribution pattern of shallow water extent in the Indo-Pacific at UTM grids shifted into different orientations. The grids were classified (equal interval) into 10 classes based on the amount of shallow water area recorded in each cell such that cells in red have the largest amount of shallow water area, and cells in blue have the lowest amount of shallow water area. (A) UTM shifted north/south, (B) UTM shifted east/west, (C) UTM shifted northeast/southwest. (PDF) [file pone.0056245.s001.pdf]
