# Supplementary material for: Habitat Availability and Heterogeneity and the Indo-Pacific Warm Pool as Predictors of Marine Species Richness in the Tropical Indo-Pacific
Source: PLoS One. 2013 Feb 15;8(2):e56245. doi: 10.1371/journal.pone.0056245 (PMC3574161; doi:10.1371/journal.pone.0056245)

Figure S5 Distribution pattern of the mean sea surface temperature in the Indo-Pacific at UTM grids shifted into different orientations.

The grids were classified (equal interval) into 10 classes based on the index values recorded in each cell such that cells in red have the highest temperature, and cells in blue have the lowest temperature. (A) UTM shifted north/south, (B) UTM shifted east/west, (C) UTM shifted northeast/southwest.

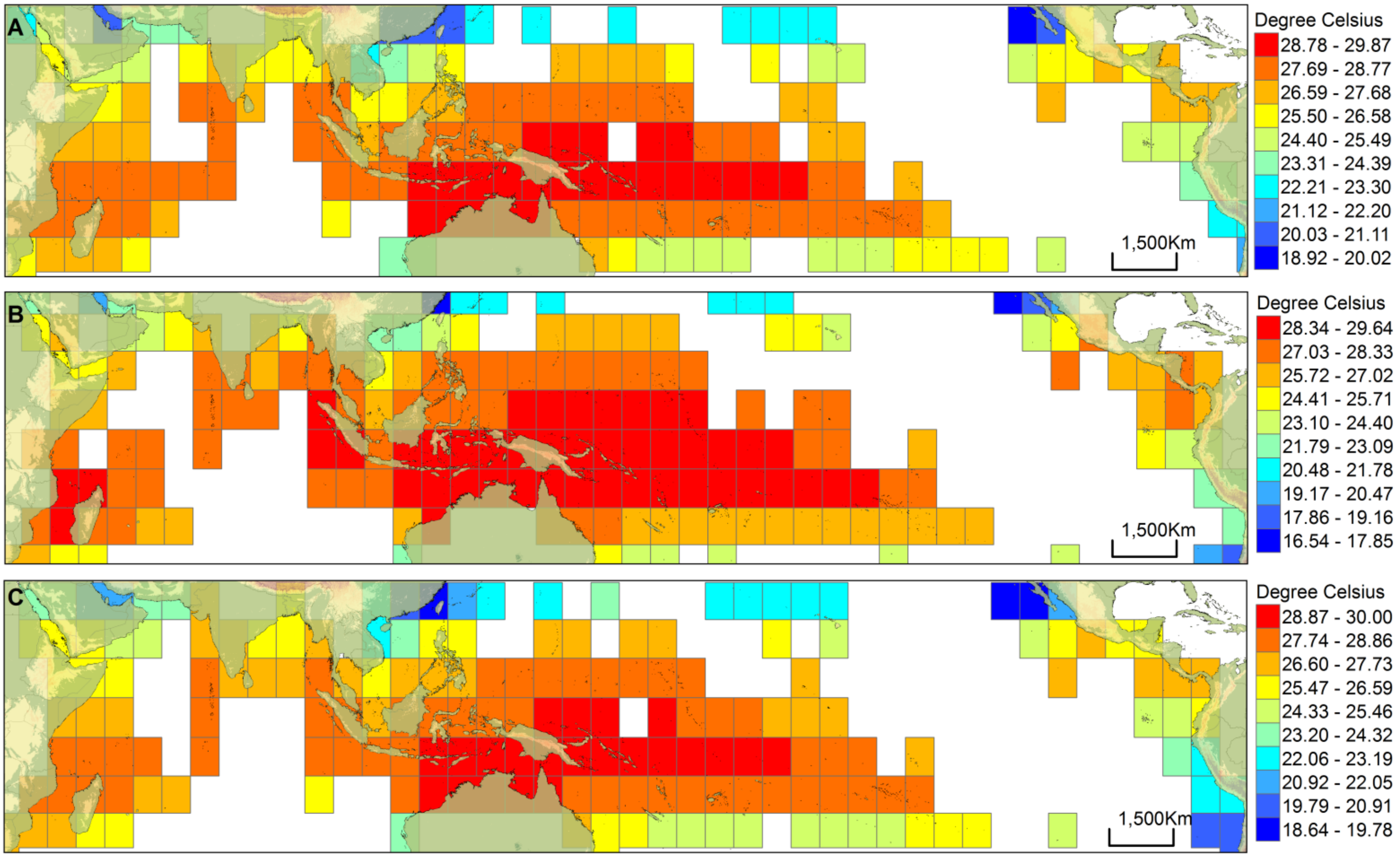

Supplement: Figure S5 — Distribution pattern of the mean sea surface temperature in the Indo-Pacific at UTM grids shifted into different orientations. The grids were classified (equal interval) into 10 classes based on the index values recorded in each cell such that cells in red have the highest temperature, and cells in blue have the lowest temperature. (A) UTM shifted north/south, (B) UTM shifted east/west, (C) UTM shifted northeast/southwest. (PDF) [file pone.0056245.s005.pdf]
