# Supplementary material for: Habitat Availability and Heterogeneity and the Indo-Pacific Warm Pool as Predictors of Marine Species Richness in the Tropical Indo-Pacific
Source: PLoS One. 2013 Feb 15;8(2):e56245. doi: 10.1371/journal.pone.0056245 (PMC3574161; doi:10.1371/journal.pone.0056245)

Figure S7 Combined adjacent cells (red color) against the regular sized cells (yellow color).

(A) Small grid, (B) Medium grid, (C) Large grid, (D) UTM, (E) UTM shifted north/south, (F) UTM shifted east/west, (G) UTM shifted northeast/southwest. There are no adjacent cells that were combined in extra large and largest grids.

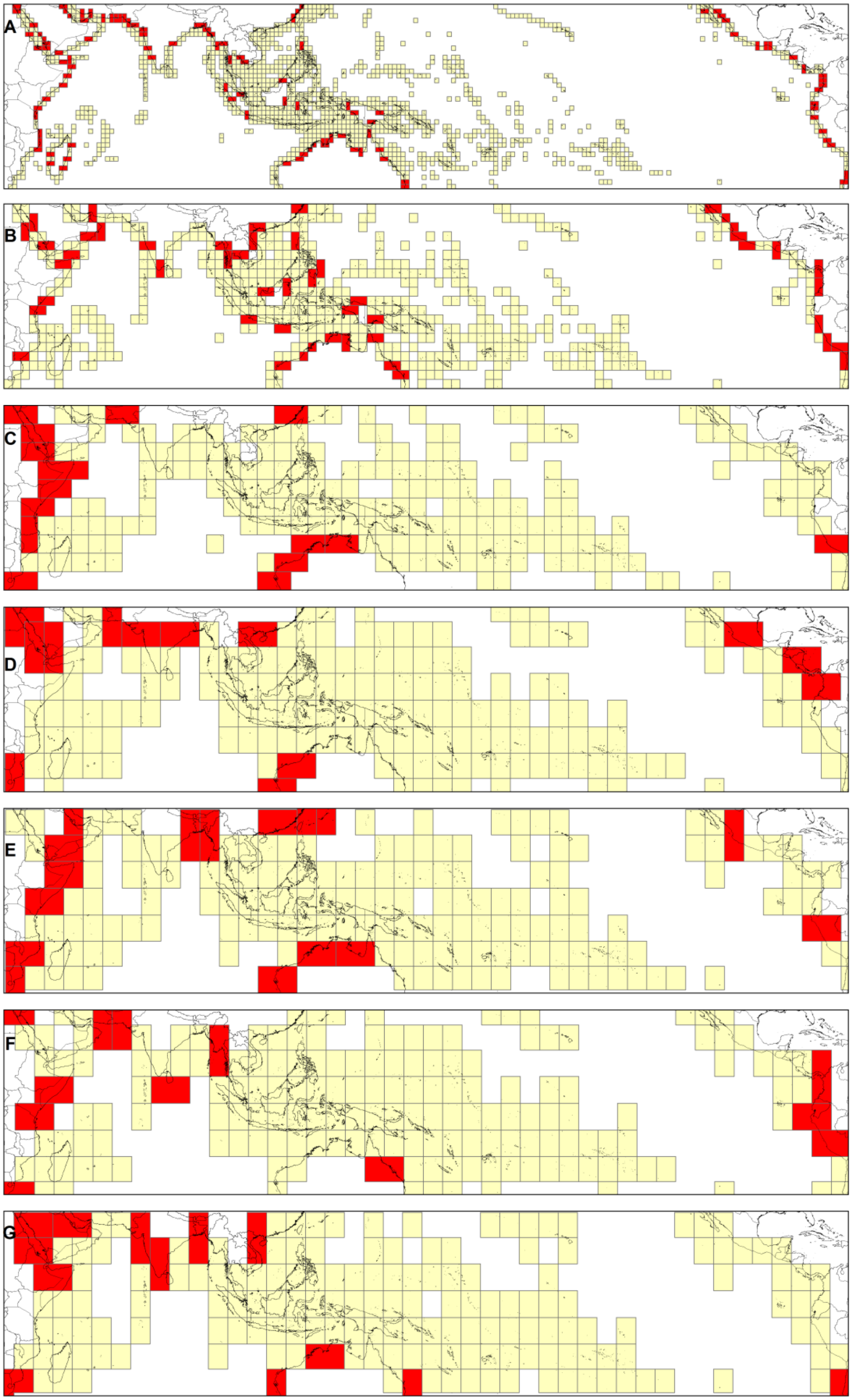

Supplement: Figure S7 — Combined adjacent cells (red color) against the regular sized cells (yellow color). (A) Small grid, (B) Medium grid, (C) Large grid, (D) UTM, (E) UTM shifted north/south, (F) UTM shifted east/west, (G) UTM shifted northeast/southwest. There are no adjacent cells that were combined in extra large and largest grids. (PDF) [file pone.0056245.s007.pdf]
