# Supplementary material for: Habitat Availability and Heterogeneity and the Indo-Pacific Warm Pool as Predictors of Marine Species Richness in the Tropical Indo-Pacific
Source: PLoS One. 2013 Feb 15;8(2):e56245. doi: 10.1371/journal.pone.0056245 (PMC3574161; doi:10.1371/journal.pone.0056245)

Figure S8 The two types of basemaps used in this study.

(A) Visualization basemap consisting of 200 m depth and 100 km buffer, (B) Bathymetry basemap showing the 200 m depth used in analyses of species richness versus independent variables.

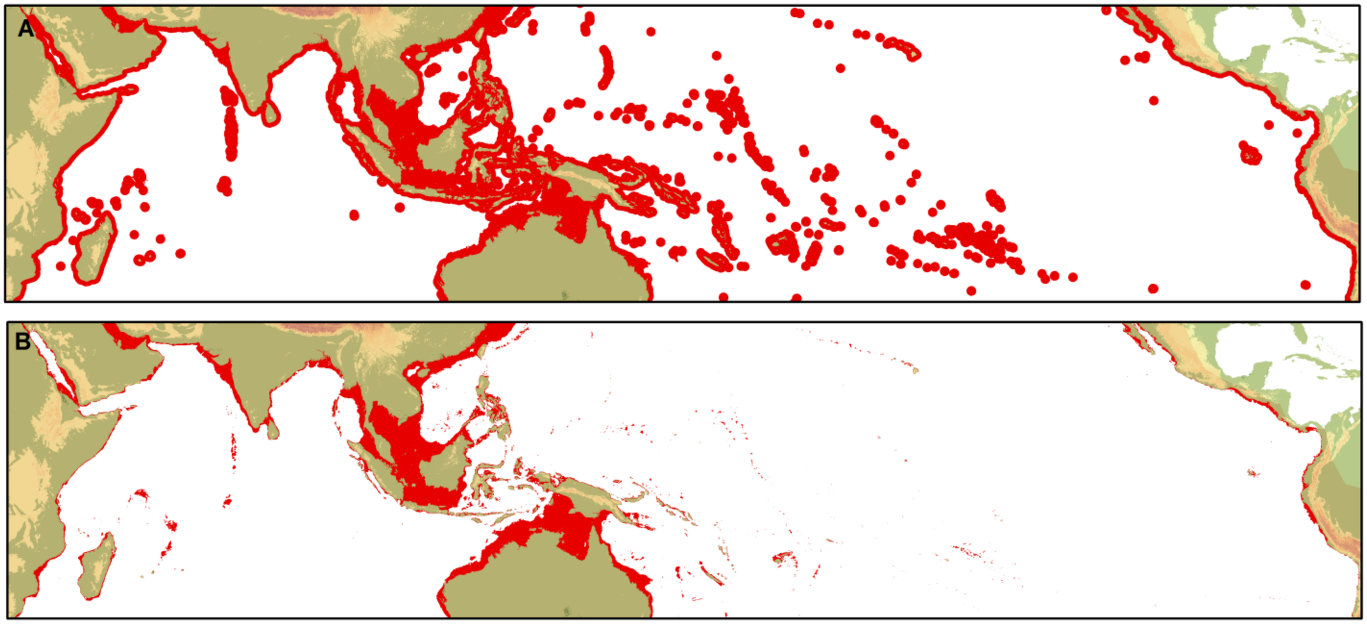

Supplement: Figure S8 — The two types of basemaps used in this study. (A) Visualization basemap consisting of 200 m depth and 100 km buffer, (B) Bathymetry basemap showing the 200 m depth used in analyses of species richness versus independent variables. (PDF) [file pone.0056245.s008.pdf]
