# Supplementary material for: Habitat Availability and Heterogeneity and the Indo-Pacific Warm Pool as Predictors of Marine Species Richness in the Tropical Indo-Pacific
Source: PLoS One. 2013 Feb 15;8(2):e56245. doi: 10.1371/journal.pone.0056245 (PMC3574161; doi:10.1371/journal.pone.0056245)

Figure S9 Distribution pattern of coastal length extent in the Indo-Pacific at different grid scales.

The grids were classified (equal interval) into 10 classes based on the amount of coastal length recorded in each cell such that cells in red have the largest amount of coastal length, and cells in blue have the lowest amount of coastal length. (A) Small grid, (B) Medium grid, (C) Large grid, (D) Extra large grid, (E) Largest grid.

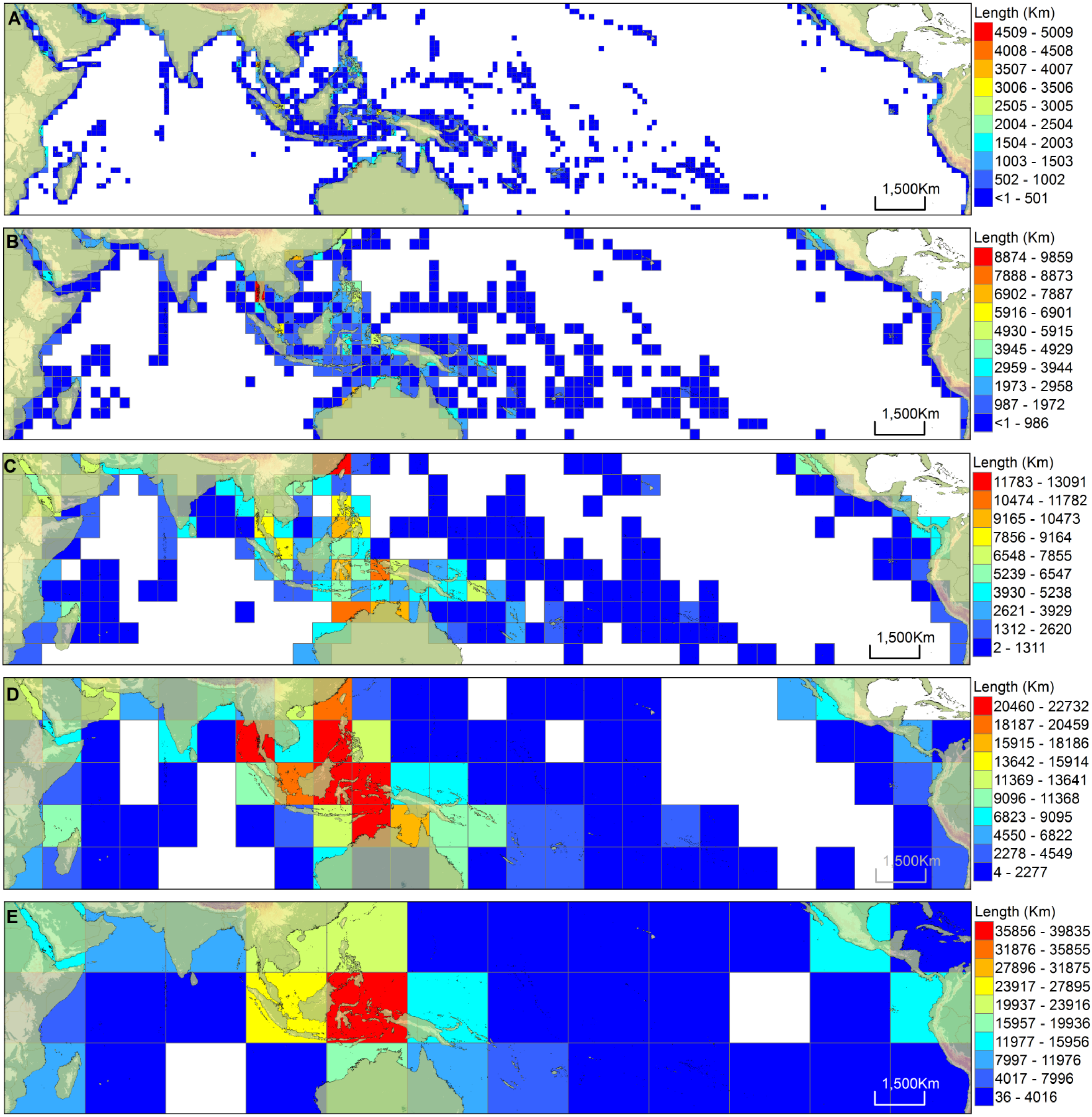

Supplement: Figure S9 — Distribution pattern of coastal length extent in the Indo-Pacific at different grid scales. The grids were classified (equal interval) into 10 classes based on the amount of coastal length recorded in each cell such that cells in red have the largest amount of coastal length, and cells in blue have the lowest amount of coastal length. (A) Small grid, (B) Medium grid, (C) Large grid, (D) Extra large grid, (E) Largest grid. (PDF) [file pone.0056245.s009.pdf]
