# Supplementary material for: Habitat Availability and Heterogeneity and the Indo-Pacific Warm Pool as Predictors of Marine Species Richness in the Tropical Indo-Pacific
Source: PLoS One. 2013 Feb 15;8(2):e56245. doi: 10.1371/journal.pone.0056245 (PMC3574161; doi:10.1371/journal.pone.0056245)

Figure S11 Distribution pattern of the habitat heterogeneity index using number in the Indo-Pacific at different grid scales.

The grids were classified (equal interval) into 10 classes based on the index values recorded in each cell such that cells in red have the largest index values, and cells in blue have the lowest index values. (A) Small grid, (B) Medium grid, (C) Large grid, (D) Extra large grid, (E) Largest grid.

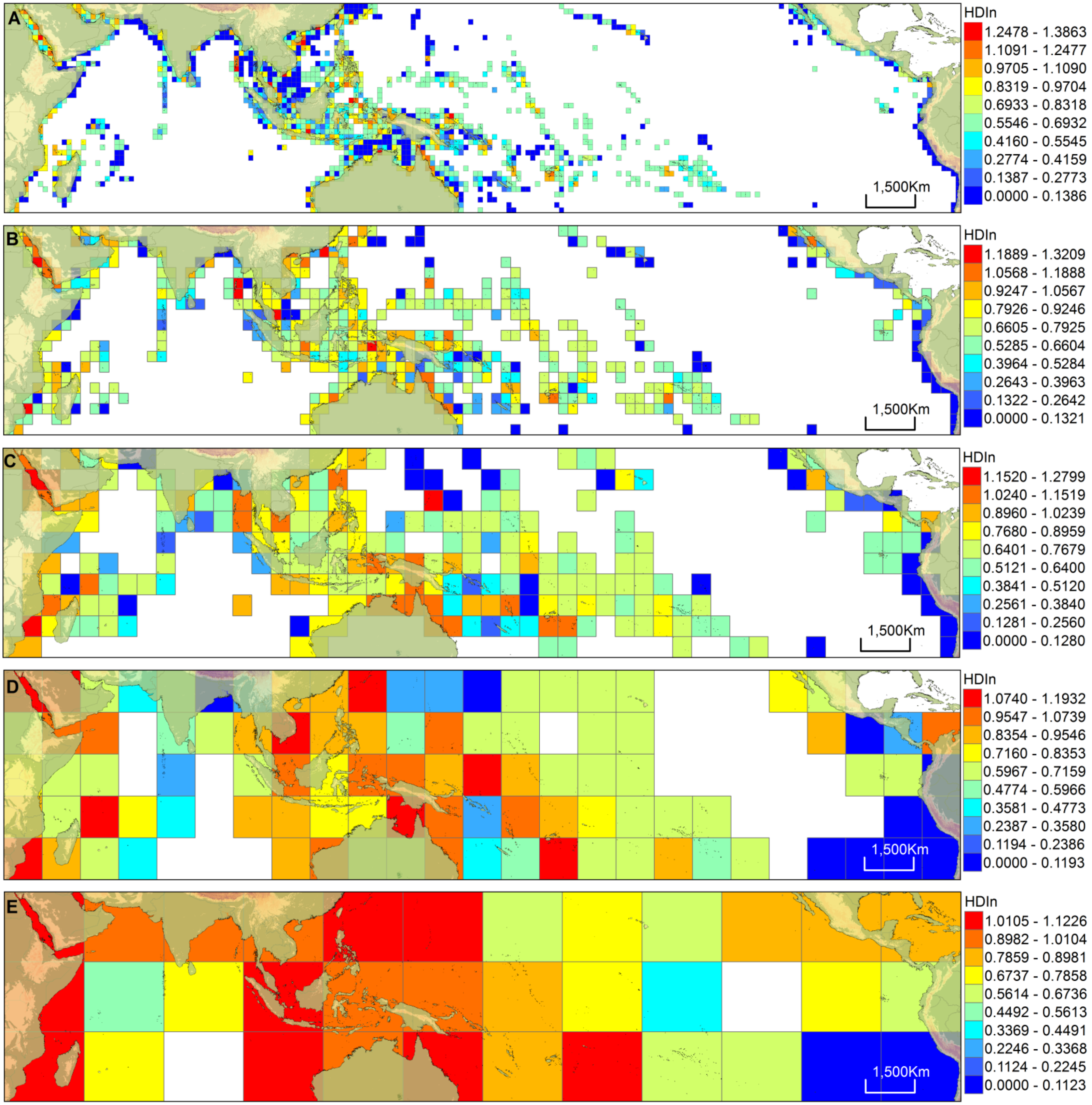

Supplement: Figure S11 — Distribution pattern of the habitat heterogeneity index using number in the Indo-Pacific at different grid scales. The grids were classified (equal interval) into 10 classes based on the index values recorded in each cell such that cells in red have the largest index values, and cells in blue have the lowest index values. (A) Small grid, (B) Medium grid, (C) Large grid, (D) Extra large grid, (E) Largest grid. (PDF) [file pone.0056245.s011.pdf]
