# Supplementary material for: Habitat Availability and Heterogeneity and the Indo-Pacific Warm Pool as Predictors of Marine Species Richness in the Tropical Indo-Pacific
Source: PLoS One. 2013 Feb 15;8(2):e56245. doi: 10.1371/journal.pone.0056245 (PMC3574161; doi:10.1371/journal.pone.0056245)
Supplement: Table S1 — Single predictor Generalized Linear Model (GLM) and Spatial Linear Model (SLM) results at different UTM grid orientations. The predictors are shallow water area (SW), coastline length (CL), habitat diversity based on area (HDIa), habitat diversity based on number of patches (HDIn), sea surface temperature (SST), and net primary productivity (NPP). Values under predictor variables are t-values for GLM and z-values for SLM. Asterisks indicate significance value of P: *(<0.05), **(<0.01); ***(<0.001); ns (not significant). UTM NS = centroid shifted on a North-South plane, UTM EW = centroid shifted on an East-West plane and UTM NE = centroid shifted along a Northeast-Southwest plane. Sample size for each grid (n) is shown. The highest adjusted R2 (GLM) and pseudo-R2 (p–R2; SLM) value within each grid size are highlighted in boldface. (PDF) [file pone.0056245.s014.pdf]

Table S1 Single predictor Generalized Linear Model (GLM) and Spatial Linear Model (SLM) results at different UTM grid orientations.

Models	Grid Size	n	SW	R^2/ p-R^2	CL	R^2/ p-R^2	HDIa	R^2/ p-R^2	HDIn	R^2/ p-R^2	SST	R^2/ p-R^2	NPP	R^2/ p-R^2
**Single predictor GLM (t-values) for all species**	UTM NS	168	8.633***	0.306	10.250***	**0.384**	2.490*	0.030	8.282***	0.288	5.303***	0.140	1.652ns	0.010
	UTM EW	173	8.479***	0.292	9.173***	**0.326**	2.214*	0.022	7.516***	0.244	6.138***	0.176	1.141ns	0.002
	UTM NE	168	9.082***	0.328	10.150***	**0.379**	2.646**	0.035	9.025***	0.325	6.483***	0.197	1.284ns	0.004
**Single predictor SLM (z-values) for all species**	UTM NS	168	6.715***	0.519	10.764***	**0.640**	3.713**	0.438	5.499***	0.482	2.801**	0.417	0.767ns	0.394
	UTM EW	173	7.631***	0.558	9.405***	**0.609**	2.808**	0.435	4.627***	0.472	4.907***	0.480	0.141ns	0.409
	UTM NE	168	7.680***	0.562	10.058***	**0.631**	3.443***	0.448	5.646***	0.500	3.720***	0.450	2.272*	0.426
**Single predictor GLM (t-values) for all fishes**	UTM NS	168	10.300***	0.386	12.040***	**0.463**	1.613ns	0.010	8.209***	0.285	4.669***	0.111	2.385*	0.027
	UTM EW	173	8.479***	0.292	10.700***	**0.398**	1.424ns	0.006	7.362***	0.236	5.431***	0.142	1.876ns	0.014
	UTM NE	168	10.810***	0.410	11.690***	**0.448**	1.575ns	0.009	8.699***	0.309	5.828***	0.165	2.209*	0.023
**Single predictor SLM (z-values) for all fishes**	UTM NS	168	7.881***	0.561	11.659***	**0.670**	3.332***	0.441	5.524***	0.493	2.428*	0.423	0.930ns	0.407
	UTM EW	173	7.878***	0.554	9.856***	**0.613**	2.464*	0.417	4.622***	0.461	4.869***	0.469	0.265ns	0.397
	UTM NE	168	8.714***	0.588	10.487***	**0.640**	2.930**	0.434	5.671***	0.498	3.609**	0.446	2.578**	0.428
**Single predictor GLM (t-values) for invertebrates**	UTM NS	168	6.002***	0.173	7.091***	0.228	3.379***	0.059	8.113***	**0.280**	6.205***	0.183	0.224ns	-0.006
	UTM EW	173	6.449***	0.191	6.126***	0.175	3.042**	0.046	7.446***	**0.240**	6.784***	0.207	-0.208ns	-0.006
	UTM NE	168	6.437***	0.195	7.277***	0.237	3.958***	0.081	9.291***	**0.338**	7.255***	0.236	-0.662ns	-0.003
**Single predictor SLM (z-values) for invertebrates**	UTM NS	168	4.427***	0.475	7.969***	**0.575**	3.863***	0.462	4.678***	0.479	3.508***	0.451	0.166ns	0.414
	UTM EW	173	5.901***	0.556	6.265***	**0.566**	2.987**	0.493	4.155***	0.514	4.355***	0.517	-0.740ns	0.469
	UTM NE	168	5.695***	0.564	8.553***	**0.637**	4.295***	0.531	5.341***	0.553	2.968**	0.502	0.999ns	0.483
**Single predictor GLM (t-values) for habitat-forming species**	UTM NS	168	6.002***	0.173	6.366***	0.191	4.459***	0.102	7.394***	**0.243**	4.886***	0.121	-0.396ns	-0.005
	UTM EW	173	4.615***	0.106	5.847***	0.162	3.898***	0.076	6.561***	**0.196**	5.590***	0.150	-0.559ns	-0.004
	UTM NE	168	4.853***	0.119	6.739***	0.210	5.441***	0.146	8.712***	**0.310**	5.874***	0.167	-1.155ns	0.002
**Single predictor SLM (z-values) for habitat-forming species**	UTM NS	168	4.109***	0.389	8.567***	**0.530**	4.092***	0.388	5.143***	0.416	2.592**	0.351	0.357ns	0.328
	UTM EW	173	5.066***	0.437	7.869***	**0.523**	3.433***	0.395	4.088***	0.409	3.573***	0.396	0.017ns	0.354
	UTM NE	168	5.248***	0.472	8.663***	**0.574**	4.637***	0.455	5.342***	0.472	2.567*	0.406	1.573ns	0.394

The predictors are shallow water area (SW), coastline length (CL), habitat diversity based on area (HDIa), habitat diversity based on number of patches (HDIn), sea surface temperature (SST), and net primary productivity (NPP). Values under predictor variables are t-values for GLM and z-values for SLM. Asterisks indicate significance value of P: * (<0.05), ** (<0.01); *** (<0.001); ns (not significant). UTM NS = centroid shifted on a North-South plane, UTM EW = centroid shifted on an East-West plane and UTM NE = centroid shifted along a Northeast-Southwest plane. Sample size for each grid (n) is shown. The highest adjusted R^2 (GLM) and pseudo-R^2 (p-R^2; SLM) value within each grid size are highlighted in boldface.
