# Supplementary material for: Habitat Availability and Heterogeneity and the Indo-Pacific Warm Pool as Predictors of Marine Species Richness in the Tropical Indo-Pacific
Source: PLoS One. 2013 Feb 15;8(2):e56245. doi: 10.1371/journal.pone.0056245 (PMC3574161; doi:10.1371/journal.pone.0056245)
Supplement: Table S2 — Multiple predictor Generalized Linear Model (GLM) and Spatial Linear Model (SLM) results at different UTM grid orientations. The predictors are shallow water area (SW), coastline length (CL), habitat diversity based on area (HDIa), habitat diversity based on number of patches (HDIn), sea surface temperature (SST), and net primary productivity (NPP). Values under predictor variables are t-values for GLM and z-values for SLM. Asterisks indicate significance value of P: *(<0.05), **(<0.01); ***(<0.001). UTM NS = centroid shifted on a North-South plane, UTM EW = centroid shifted on an East-West plane and UTM NE = centroid shifted along a Northeast-Southwest plane. Sample size for each grid (n) is shown. R2 values are for GLM results, while pseudo-R2 (p–R2) values are for SLM results. (PDF) [file pone.0056245.s015.pdf]

Table S2 Multiple predictor Generalized Linear Model (GLM) and Spatial Linear Model (SLM) results at different UTM grid orientations.

Models	Grid Size	n	SW	CL	HDIa	HDIn	SST	NPP	Moran's *I*	R^2/ p-R^2	AIC
**Single predictor GLM (t-values) for all species**	UTM NS	168	2.959**	3.787***	2.478*	3.356***	6.112***		0.2590***	0.604	288.39
	UTM EW	173	3.317**	2.585*		3.721***	7.331***		0.3359***	0.557	316.58
	UTM NE	168	4.910***	2.224*	4.240***	3.384***	5.585***		0.1744***	0.664	260.13
**Single predictor SLM (z-values) for all species**	UTM NS	168		9.657***		2.988**	3.934***		-0.0223	0.683	254.17
	UTM EW	173		9.939***			5.705***		-0.0091	0.669	268.57
	UTM NE	168	2.668**	4.204***	4.045***	4.677***	2.852**		-0.0137	0.715	239.56
**Single predictor GLM (t-values) for all fishes**	UTM NS	168	2.774**	5.204***		4.364***	6.724***		0.2630***	0.637	240.48
	UTM EW	173	3.339**	3.548***		3.312**	6.873***		0.3067***	0.586	276.54
	UTM NE	168	4.938***	2.696**	3.993***	3.544***	5.660***	2.400*	0.1758***	0.694	210.96
**Single predictor SLM (z-values) for all fishes**	UTM NS	168		10.605***		2.970**	3.706***		-0.0222	0.707	208.33
	UTM EW	173		10.542***			5.821***		-0.0068	0.675	236.51
	UTM NE	168	3.373***	3.683***	3.004**	2.143*	5.010***	3.068**	-0.0094	0.737	193.95
**Single predictor GLM (t-values) for invertebrates**	UTM NS	168		5.123***	3.136**	3.660***	4.240***		0.3467***	0.426	494.49
	UTM EW	173	4.804***		2.888**	2.499*	4.185***		0.3277***	0.362	529.48
	UTM NE	168	6.672***		6.361***	3.422***	3.073**		0.2099***	0.531	468.31
**Single predictor SLM (z-values) for invertebrates**	UTM NS	168	-3.055**	7.287***		2.758**	2.436*		-0.0215	0.591	443.64
	UTM EW	173		7.842***			3.523***		-0.0222	0.553	470.23
	UTM NE	168		8.532***	4.500***		2.388*	2.163*	-0.0305	0.64	430.22
**Single predictor GLM (t-values) for habitat-forming species**	UTM NS	168	2.034*	2.182*	2.256*	3.993***	6.277***		0.2632***	0.513	533.22
	UTM EW	173	6.576***		2.109*	3.205**	6.292***		0.3240***	0.492	543.43
	UTM NE	168	7.580***		4.869***	4.103***	5.446***		0.2297***	0.596	507.25
**Single predictor SLM (z-values) for habitat-forming species**	UTM NS	168		7.952***	2.668**		3.649***		0.0022	0.626	491.89
	UTM EW	173	6.773***		0.921**		4.061***		0.0017	0.625	495.24
	UTM NE	168		8.589***	3.640***		2.811**		-0.0126	0.682	470.92

The predictors are shallow water area (SW), coastline length (CL), habitat diversity based on area (HDIa), habitat diversity based on number of patches (HDIn), sea surface temperature (SST), and net primary productivity (NPP). Values under predictor variables are t-values for GLM and z-values for SLM. Asterisks indicate significance value of P: * (<0.05), ** (<0.01); *** (<0.001). UTM NS = centroid shifted on a North-South plane, UTM EW = centroid shifted on an East-West plane and UTM NE = centroid shifted along a Northeast-Southwest plane. Sample size for each grid (n) is shown. R^2 values are for GLM results, while pseudo-R^2 (p-R^2) values are for SLM results.
