# Supplementary material for: Habitat Availability and Heterogeneity and the Indo-Pacific Warm Pool as Predictors of Marine Species Richness in the Tropical Indo-Pacific
Source: PLoS One. 2013 Feb 15;8(2):e56245. doi: 10.1371/journal.pone.0056245 (PMC3574161; doi:10.1371/journal.pone.0056245)
Supplement: Table S3 — List of families with the number of species distribution maps used in this study. (PDF) [file pone.0056245.s016.pdf]

Table S3 List of families with the number of species distribution maps used in this study.

Kingdom	*Group*	*Family*	*Number of Species*
**Plantae**	Mangroves	Acanthaceae	11
**Plantae**	Mangroves	Arecaceae	2
**Plantae**	Mangroves	Bignoniaceae	2
**Plantae**	Mangroves	Combretaceae	4
**Plantae**	Mangroves	Ebenaceae	1
**Plantae**	Mangroves	Euphorbiaceae	2
**Plantae**	Mangroves	Fabaceae	2
**Plantae**	Mangroves	Lythraceae	7
**Plantae**	Mangroves	Malvaceae	7
**Plantae**	Mangroves	Meliaceae	3
**Plantae**	Mangroves	Myrsinaceae	2
**Plantae**	Mangroves	Myrtaceae	1
**Plantae**	Mangroves	Plumbaginaceae	2
**Plantae**	Mangroves	Pteridaceae	3
**Plantae**	Mangroves	Rhizophoraceae	18
**Plantae**	Mangroves	Rubiaceae	1
**Plantae**	Mangroves	Tetrameristaceae	1
**Plantae**	Seagrasses	Cymodoceaceae	13
**Plantae**	Seagrasses	Hydrocharitaceae	19
**Plantae**	Seagrasses	Posidoniaceae	4
**Plantae**	Seagrasses	Ruppiaceae	3
**Plantae**	Seagrasses	Zannichelliaceae	1
**Plantae**	Seagrasses	Zosteraceae	10
**Animalia**	Corals	Acroporidae	269
**Animalia**	Corals	Agariciidae	45
**Animalia**	Corals	Astrocoeniidae	13
**Animalia**	Corals	Caryophylliidae	3
**Animalia**	Corals	Dendrophylliidae	14
**Animalia**	Corals	Euphyllidae	16
**Animalia**	Corals	Faviidae	128
**Animalia**	Corals	Fungiidae	46
**Animalia**	Corals	Helioporidae	1
**Animalia**	Corals	Meandrinidae	9
**Animalia**	Corals	Merulinidae	12
**Animalia**	Corals	Milleporidae	13
**Animalia**	Corals	Mussidae	49
**Animalia**	Corals	Oculinidae	11
**Animalia**	Corals	Pectinidae	29
**Animalia**	Corals	Pocilloporidae	31
**Animalia**	Corals	Poritidae	99
**Animalia**	Corals	Siderastreidae	30
**Animalia**	Corals	Trachyphylliidae	1
**Animalia**	Corals	Tubiporidae	1
**Animalia**	Molluscs	Actinocyclidae	2
**Animalia**	Molluscs	Bursidae	20
**Animalia**	Molluscs	Callochitonidae	15
**Animalia**	Molluscs	Cassidae	21

**Animalia**	Molluscs	Cerithiidae	8
**Animalia**	Molluscs	Chaetopleuridae	1
**Animalia**	Molluscs	Condylocardiidae	12
**Animalia**	Molluscs	Cypraeidae	187
**Animalia**	Molluscs	Haliotidae	46
**Animalia**	Molluscs	Harpidae	14
**Animalia**	Molluscs	Hemiarthridae	3
**Animalia**	Molluscs	Ischnochitonidae	39
**Animalia**	Molluscs	Leptochitonidae	19
**Animalia**	Molluscs	Littorinidae	46
**Animalia**	Molluscs	Mitridae	180
**Animalia**	Molluscs	Muricidae	9
**Animalia**	Molluscs	Olividae	114
**Animalia**	Molluscs	Patellidae	40
**Animalia**	Molluscs	Personidae	15
**Animalia**	Molluscs	Phasianellidae	2
**Animalia**	Molluscs	Pinnidae	7
**Animalia**	Molluscs	Ranellidae	48
**Animalia**	Molluscs	Strombidae	50
**Animalia**	Molluscs	Tonicellidae	14
**Animalia**	Molluscs	Tridacnidae	6
**Animalia**	Molluscs	Triviidae	18
**Animalia**	Molluscs	Trochidae	6
**Animalia**	Molluscs	Vasidae	13
**Animalia**	Crustaceans	Diogenidae	19
**Animalia**	Crustaceans	Dynomenidae	10
**Animalia**	Crustaceans	Eurysquillidae	5
**Animalia**	Crustaceans	Gonodactylidae	20
**Animalia**	Crustaceans	Harpiosquillidae	5
**Animalia**	Crustaceans	Heterosquillidae	1
**Animalia**	Crustaceans	Homolidae	44
**Animalia**	Crustaceans	Homolodromiidae	10
**Animalia**	Crustaceans	Leucosiidae	26
**Animalia**	Crustaceans	Lysiosquillidae	3
**Animalia**	Crustaceans	Nannosquillidae	3
**Animalia**	Crustaceans	Odontodactylidae	2
**Animalia**	Crustaceans	Portunidae	35
**Animalia**	Crustaceans	Protosquillidae	6
**Animalia**	Crustaceans	Pseudosquillidae	2
**Animalia**	Crustaceans	Rhynchocinetidae	6
**Animalia**	Crustaceans	Squillidae	31
**Animalia**	Crustaceans	Takuidae	1
**Animalia**	Crustaceans	Trapeziidae	28
**Animalia**	Chimaeras	Callorhinchidae	2
**Animalia**	Chimaeras	Chimaeridae	15
**Animalia**	Chimaeras	Rhinochimaeridae	5
**Animalia**	Sharks	Alopiidae	3
**Animalia**	Sharks	Brachaeluridae	2
**Animalia**	Sharks	Carcharhinidae	51

**Animalia**	Sharks	Centrophoridae	15
**Animalia**	Sharks	Cetorhinidae	1
**Animalia**	Sharks	Chlamydoselachidae	1
**Animalia**	Sharks	Dalatiidae	9
**Animalia**	Sharks	Echinorhinidae	2
**Animalia**	Sharks	Etmopteridae	35
**Animalia**	Sharks	Ginglymostomatidae	3
**Animalia**	Sharks	Hemigaleidae	7
**Animalia**	Sharks	Hemiscylliidae	12
**Animalia**	Sharks	Heterodontidae	9
**Animalia**	Sharks	Hexanchidae	4
**Animalia**	Sharks	Lamnidae	5
**Animalia**	Sharks	Megachasmidae	1
**Animalia**	Sharks	Mitsukurinidae	1
**Animalia**	Sharks	Odontaspididae	3
**Animalia**	Sharks	Orectolobidae	10
**Animalia**	Sharks	Oxynotidae	1
**Animalia**	Sharks	Parascylliidae	5
**Animalia**	Sharks	Proscylliidae	5
**Animalia**	Sharks	Pseudocarchariidae	1
**Animalia**	Sharks	Pseudotriakidae	16
**Animalia**	Sharks	Rhincodontidae	1
**Animalia**	Sharks	Scyliorhinidae	107
**Animalia**	Sharks	Somniosidae	9
**Animalia**	Sharks	Sphyrnidae	8
**Animalia**	Sharks	Squalidae	23
**Animalia**	Sharks	Squatinidae	13
**Animalia**	Sharks	Stegostomatidae	2
**Animalia**	Sharks	Triakidae	37
**Animalia**	Batoid Fishes	Anacanthobatidae	23
**Animalia**	Batoid Fishes	Arhynchobatidae	14
**Animalia**	Batoid Fishes	Dasyatidae	55
**Animalia**	Batoid Fishes	Gymnuridae	9
**Animalia**	Batoid Fishes	Hexatrygonidae	1
**Animalia**	Batoid Fishes	Hypnidae	1
**Animalia**	Batoid Fishes	Mobulidae	8
**Animalia**	Batoid Fishes	Myliobatidae	17
**Animalia**	Batoid Fishes	Narcinidae	24
**Animalia**	Batoid Fishes	Narkidae	9
**Animalia**	Batoid Fishes	Platyrhinidae	3
**Animalia**	Batoid Fishes	Plesiobatidae	1
**Animalia**	Batoid Fishes	Pristidae	6
**Animalia**	Batoid Fishes	Pristiophoridae	6
**Animalia**	Batoid Fishes	Rajidae	62
**Animalia**	Batoid Fishes	Rhinidae	1
**Animalia**	Batoid Fishes	Rhinobatidae	37
**Animalia**	Batoid Fishes	Rhinopteridae	5
**Animalia**	Batoid Fishes	Rhynchobatidae	3
**Animalia**	Batoid Fishes	Torpedinidae	13

**Animalia**	Batoid Fishes	Urolophidae	20
**Animalia**	Batoid Fishes	Urotrygonidae	13
**Animalia**	Bony Fishes	Acanthuridae	75
**Animalia**	Bony Fishes	Achiridae	8
**Animalia**	Bony Fishes	Acropomatidae	16
**Animalia**	Bony Fishes	Albulidae	11
**Animalia**	Bony Fishes	Amarsipidae	1
**Animalia**	Bony Fishes	Ambassidae	16
**Animalia**	Bony Fishes	Ammodytidae	9
**Animalia**	Bony Fishes	Anablepidae	2
**Animalia**	Bony Fishes	Anguillidae	10
**Animalia**	Bony Fishes	Anomalopidae	4
**Animalia**	Bony Fishes	Antennariidae	33
**Animalia**	Bony Fishes	Apistidae	1
**Animalia**	Bony Fishes	Aploactinidae	37
**Animalia**	Bony Fishes	Apogonidae	249
**Animalia**	Bony Fishes	Argentinidae	2
**Animalia**	Bony Fishes	Ariidae	73
**Animalia**	Bony Fishes	Ariommatidae	2
**Animalia**	Bony Fishes	Arripidae	4
**Animalia**	Bony Fishes	Atherinidae	32
**Animalia**	Bony Fishes	Atherinopsidae	17
**Animalia**	Bony Fishes	Aulopidae	1
**Animalia**	Bony Fishes	Aulostomidae	1
**Animalia**	Bony Fishes	Balistidae	31
**Animalia**	Bony Fishes	Batrachoididae	30
**Animalia**	Bony Fishes	Belonidae	25
**Animalia**	Bony Fishes	Bembridae	3
**Animalia**	Bony Fishes	Berycidae	7
**Animalia**	Bony Fishes	Blenniidae	299
**Animalia**	Bony Fishes	Bothidae	113
**Animalia**	Bony Fishes	Brachionochthyidae	1
**Animalia**	Bony Fishes	Bramidae	11
**Animalia**	Bony Fishes	Branchiostegidae	12
**Animalia**	Bony Fishes	Bregmacerotidae	9
**Animalia**	Bony Fishes	Bythitidae	89
**Animalia**	Bony Fishes	Caesionidae	20
**Animalia**	Bony Fishes	Callanthiidae	7
**Animalia**	Bony Fishes	Callionymidae	139
**Animalia**	Bony Fishes	Caproidae	5
**Animalia**	Bony Fishes	Caracanthidae	4
**Animalia**	Bony Fishes	Carangidae	102
**Animalia**	Bony Fishes	Carapidae	22
**Animalia**	Bony Fishes	Centracanthidae	2
**Animalia**	Bony Fishes	Centriscidae	8
**Animalia**	Bony Fishes	Centrogeniidae	2
**Animalia**	Bony Fishes	Centrolophidae	6
**Animalia**	Bony Fishes	Centropomidae	7
**Animalia**	Bony Fishes	Cepolidae	4

**Animalia**	Bony Fishes	Chaenopsidae	32
**Animalia**	Bony Fishes	Chaetodontidae	112
**Animalia**	Bony Fishes	Chanidae	1
**Animalia**	Bony Fishes	Cheilodactylidae	17
**Animalia**	Bony Fishes	Chirocentridae	2
**Animalia**	Bony Fishes	Chlopsidae	10
**Animalia**	Bony Fishes	Cichlidae	1
**Animalia**	Bony Fishes	Cirrhitidae	29
**Animalia**	Bony Fishes	Citharidae	4
**Animalia**	Bony Fishes	Clinidae	3
**Animalia**	Bony Fishes	Clupeidae	86
**Animalia**	Bony Fishes	Congridae	48
**Animalia**	Bony Fishes	Coryphaenidae	2
**Animalia**	Bony Fishes	Creediidae	10
**Animalia**	Bony Fishes	Cynoglossidae	78
**Animalia**	Bony Fishes	Cyttidae	1
**Animalia**	Bony Fishes	Dactylopteridae	6
**Animalia**	Bony Fishes	Dactyloscopidae	23
**Animalia**	Bony Fishes	Dentatherinidae	1
**Animalia**	Bony Fishes	Dinopercidae	1
**Animalia**	Bony Fishes	Diodontidae	11
**Animalia**	Bony Fishes	Drepanidae	2
**Animalia**	Bony Fishes	Echeneidae	7
**Animalia**	Bony Fishes	Eleotridae	41
**Animalia**	Bony Fishes	Elopidae	2
**Animalia**	Bony Fishes	Embiotocidae	1
**Animalia**	Bony Fishes	Engraulidae	92
**Animalia**	Bony Fishes	Enoplosidae	1
**Animalia**	Bony Fishes	Ephippidae	12
**Animalia**	Bony Fishes	Epigonidae	1
**Animalia**	Bony Fishes	Exocoetidae	51
**Animalia**	Bony Fishes	Fistulariidae	3
**Animalia**	Bony Fishes	Gempylidae	4
**Animalia**	Bony Fishes	Gerreidae	34
**Animalia**	Bony Fishes	Girellidae	2
**Animalia**	Bony Fishes	Glaucosomatidae	4
**Animalia**	Bony Fishes	Gobiesocidae	62
**Animalia**	Bony Fishes	Gobiidae	848
**Animalia**	Bony Fishes	Gonorynchidae	5
**Animalia**	Bony Fishes	Haemulidae	97
**Animalia**	Bony Fishes	Hemiramphidae	49
**Animalia**	Bony Fishes	Heterenchelyidae	1
**Animalia**	Bony Fishes	Holocentridae	67
**Animalia**	Bony Fishes	Hoplichthyidae	7
**Animalia**	Bony Fishes	Isonidae	3
**Animalia**	Bony Fishes	Istiophoridae	5
**Animalia**	Bony Fishes	Kraemeriidae	8
**Animalia**	Bony Fishes	Kuhliidae	10
**Animalia**	Bony Fishes	Kurtidae	2

**Animalia**	Bony Fishes	Kyphosidae	38
**Animalia**	Bony Fishes	Labridae	471
**Animalia**	Bony Fishes	Labrisomidae	42
**Animalia**	Bony Fishes	Lactariidae	1
**Animalia**	Bony Fishes	Lampridae	2
**Animalia**	Bony Fishes	Latidae	1
**Animalia**	Bony Fishes	Latimeriidae	1
**Animalia**	Bony Fishes	Leiognathidae	43
**Animalia**	Bony Fishes	Lepisosteidae	1
**Animalia**	Bony Fishes	Leptobramidae	1
**Animalia**	Bony Fishes	Leptoscopidae	1
**Animalia**	Bony Fishes	Lethrinidae	37
**Animalia**	Bony Fishes	Lobotidae	4
**Animalia**	Bony Fishes	Lophichthyidae	1
**Animalia**	Bony Fishes	Lophiidae	6
**Animalia**	Bony Fishes	Lutjanidae	82
**Animalia**	Bony Fishes	Luvaridae	1
**Animalia**	Bony Fishes	Macrorhamphosidae	1
**Animalia**	Bony Fishes	Malacanthidae	16
**Animalia**	Bony Fishes	Megalopidae	2
**Animalia**	Bony Fishes	Menidae	1
**Animalia**	Bony Fishes	Merlucciidae	3
**Animalia**	Bony Fishes	Microcanthidae	1
**Animalia**	Bony Fishes	Microdesmidae	23
**Animalia**	Bony Fishes	Molidae	4
**Animalia**	Bony Fishes	Monacanthidae	64
**Animalia**	Bony Fishes	Monocentridae	2
**Animalia**	Bony Fishes	Monodactylidae	5
**Animalia**	Bony Fishes	Moridae	17
**Animalia**	Bony Fishes	Moringuidae	7
**Animalia**	Bony Fishes	Mugilidae	53
**Animalia**	Bony Fishes	Mullidae	55
**Animalia**	Bony Fishes	Muraenesocidae	6
**Animalia**	Bony Fishes	Muraenidae	140
**Animalia**	Bony Fishes	Myrocongridae	1
**Animalia**	Bony Fishes	Nematistiidae	1
**Animalia**	Bony Fishes	Nemipteridae	65
**Animalia**	Bony Fishes	Neosebastidae	6
**Animalia**	Bony Fishes	Nettastomatidae	3
**Animalia**	Bony Fishes	Nomeidae	12
**Animalia**	Bony Fishes	Ogcocephalidae	10
**Animalia**	Bony Fishes	Ophichthidae	137
**Animalia**	Bony Fishes	Ophidiidae	25
**Animalia**	Bony Fishes	Opisthognathidae	67
**Animalia**	Bony Fishes	Oplegnathidae	1
**Animalia**	Bony Fishes	Ostraciidae	14
**Animalia**	Bony Fishes	Parabembridae	1
**Animalia**	Bony Fishes	Paralichthyidae	59
**Animalia**	Bony Fishes	Pegasidae	5

**Animalia**	Bony Fishes	Pempheridae	21
**Animalia**	Bony Fishes	Pentacerotidae	7
**Animalia**	Bony Fishes	Percophidae	9
**Animalia**	Bony Fishes	Peristediidae	5
**Animalia**	Bony Fishes	Phallostethidae	3
**Animalia**	Bony Fishes	Pholidichthyidae	2
**Animalia**	Bony Fishes	Pinguipedidae	47
**Animalia**	Bony Fishes	Platycephalidae	68
**Animalia**	Bony Fishes	Plesiopidae	40
**Animalia**	Bony Fishes	Pleuronectidae	27
**Animalia**	Bony Fishes	Plotosidae	13
**Animalia**	Bony Fishes	Poeciliidae	5
**Animalia**	Bony Fishes	Polynemidae	29
**Animalia**	Bony Fishes	Polyprionidae	1
**Animalia**	Bony Fishes	Pomacanthidae	80
**Animalia**	Bony Fishes	Pomacentridae	327
**Animalia**	Bony Fishes	Pomatomidae	1
**Animalia**	Bony Fishes	Priacanthidae	16
**Animalia**	Bony Fishes	Pristigasteridae	15
**Animalia**	Bony Fishes	Psettodidae	1
**Animalia**	Bony Fishes	Pseudochromidae	141
**Animalia**	Bony Fishes	Pseudomugilidae	3
**Animalia**	Bony Fishes	Ptereleotridae	40
**Animalia**	Bony Fishes	Rachycentridae	1
**Animalia**	Bony Fishes	Samaridae	1
**Animalia**	Bony Fishes	Scatophagidae	4
**Animalia**	Bony Fishes	Schindleriidae	3
**Animalia**	Bony Fishes	Sciaenidae	184
**Animalia**	Bony Fishes	Scombridae	38
**Animalia**	Bony Fishes	Scombrolabracidae	1
**Animalia**	Bony Fishes	Scorpaenidae	167
**Animalia**	Bony Fishes	Scorpididae	1
**Animalia**	Bony Fishes	Sebastidae	1
**Animalia**	Bony Fishes	Serranidae	352
**Animalia**	Bony Fishes	Setarchidae	2
**Animalia**	Bony Fishes	Siganidae	28
**Animalia**	Bony Fishes	Sillaginidae	29
**Animalia**	Bony Fishes	Soleidae	99
**Animalia**	Bony Fishes	Solenostomidae	5
**Animalia**	Bony Fishes	Sparidae	70
**Animalia**	Bony Fishes	Sphyraenidae	18
**Animalia**	Bony Fishes	Stromateidae	9
**Animalia**	Bony Fishes	Symphysanodontidae	5
**Animalia**	Bony Fishes	Synanceiidae	26
**Animalia**	Bony Fishes	Syngnathidae	203
**Animalia**	Bony Fishes	Synodontidae	46
**Animalia**	Bony Fishes	Telmatherinidae	1
**Animalia**	Bony Fishes	Terapontidae	14
**Animalia**	Bony Fishes	Tetrabrachiidae	1

**Animalia**	Bony Fishes	Tetragonuridae	3
**Animalia**	Bony Fishes	Tetraodontidae	45
**Animalia**	Bony Fishes	Tetrarogidae	15
**Animalia**	Bony Fishes	Toxotidae	2
**Animalia**	Bony Fishes	Trachichthyidae	5
**Animalia**	Bony Fishes	Triacanthidae	7
**Animalia**	Bony Fishes	Trichiuridae	7
**Animalia**	Bony Fishes	Trichonotidae	8
**Animalia**	Bony Fishes	Triglidae	57
**Animalia**	Bony Fishes	Tripterygiidae	96
**Animalia**	Bony Fishes	Uranoscopidae	30
**Animalia**	Bony Fishes	Urolophidae	33
**Animalia**	Bony Fishes	Veliferidae	2
**Animalia**	Bony Fishes	Xenisthmidae	13
**Animalia**	Bony Fishes	Xiphiidae	1
**Animalia**	Bony Fishes	Zanclidae	1
**Animalia**	Bony Fishes	Zeidae	4
