# Supplementary material for: Habitat Availability and Heterogeneity and the Indo-Pacific Warm Pool as Predictors of Marine Species Richness in the Tropical Indo-Pacific
Source: PLoS One. 2013 Feb 15;8(2):e56245. doi: 10.1371/journal.pone.0056245 (PMC3574161; doi:10.1371/journal.pone.0056245)
Supplement: Table S4 — Single predictor Generalized Linear Model (GLM) and Spatial Linear Model (SLM) complete results at different grid sizes. The predictors are shallow water area (SW), coastline length (CL), habitat diversity based on area (HDIa), habitat diversity based on number of patches (HDIn), sea surface temperature (SST), and net primary productivity (NPP). Values under predictor variables are t-values for GLM and z-values for SLM. Asterisks indicate significance value of P: *(<0.05), **(<0.01); ***(<0.001); ns (not significant). Sample size for each grid (n) is shown. The highest adjusted R2 (GLM) and pseudo-R2 (p–R2; SLM) value within each grid size are highlighted in boldface. (PDF) [file pone.0056245.s017.pdf]

Table S4 Single predictor Generalized Linear Model (GLM) and Spatial Linear Model (SLM) complete results at different grid sizes.

Models	Grid Size	n	SW	R^2/ p-R^2	CL	R^2/ p-R^2	HDIa	R^2/ p-R^2	HDIn	R^2/ p-R^2	SST	R^2/ p-R^2	NPP	R^2/ p-R^2
**Single predictor GLM (t-values) for all species**	Small	1390	13.930***	0.122	16.080***	**0.157**	4.254***	0.012	10.290***	0.070	0.790ns	0.000	3.599***	0.009
	Medium	570	11.940***	0.199	16.390***	**0.320**	3.587***	0.020	9.704***	0.141	2.073*	0.006	2.284*	0.007
	Large	223	8.959***	0.263	11.490***	**0.371**	2.926**	0.033	8.720***	0.253	5.173***	0.104	1.665ns	0.008
	UTM	175	8.604***	0.296	11.220***	**0.418**	2.609**	0.032	7.931***	0.262	6.557***	0.194	0.416ns	-0.005
	X Large	95	6.861***	0.329	10.040***	**0.515**	2.648**	0.060	8.916***	0.455	6.425***	0.300	0.848ns	-0.003
	Largest	34	6.281***	0.538	5.296***	**0.451**	2.792**	0.171	6.356***	0.544	4.346***	0.352	0.480ns	-0.024
**Single predictor SLM (z-values) for all species**	Small	1390	11.562***	**0.494**	10.183***	0.484	3.566***	0.019	4.302***	0.453	0.161ns	0.446	1.985*	0.448
	Medium	570	10.486***	0.444	12.407***	**0.474**	3.406***	0.351	5.506***	0.370	0.801ns	0.339	1.004ns	0.339
	Large	223	7.507***	0.415	11.125***	**0.530**	4.854***	0.338	6.326***	0.376	3.934***	0.315	1.170ns	0.274
	UTM	175	7.618***	0.495	10.615***	**0.591**	2.530*	0.352	5.459***	0.423	4.043***	0.382	0.145ns	0.329
	X Large	95	5.221***	0.635	10.047***	**0.773**	1.579ns	0.544	3.974***	0.593	3.014**	0.569	0.825ns	0.535
	Largest	34	4.480***	0.754	5.470***	**0.793**	0.655ns	0.616	3.241**	0.697	2.573*	0.675	1.605ns	0.639
**Single predictor GLM (t-values) for all fishes**	Small	1390	14.960***	0.138	17.410***	**0.179**	3.768***	0.009	10.410***	0.072	0.278ns	-0.001	4.514***	0.014
	Medium	570	13.570***	0.243	17.910***	**0.360**	3.006**	0.014	9.756***	0.142	1.804ns	0.004	3.209**	0.016
	Large	223	10.610***	0.334	13.340***	**0.444**	1.767ns	0.009	8.638***	0.249	4.463***	0.079	2.820**	0.030
	UTM	175	9.906***	0.358	13.780***	**0.521**	1.988*	0.017	8.142***	0.273	5.738***	0.155	1.188ns	0.002
	X Large	95	6.861***	0.329	12.290***	**0.615**	1.491ns	0.013	8.583***	0.436	5.857***	0.262	1.908ns	0.027
	Largest	34	7.440***	0.622	6.401***	**0.548**	2.671*	0.157	6.361***	0.545	3.840***	0.294	0.890ns	-0.006
**Single predictor SLM (z-values) for all fishes**	Small	1390	12.522***	**0.518**	10.425***	0.502	3.391***	0.468	4.270***	0.470	0.185ns	0.463	2.105*	0.465
	Medium	570	11.515***	0.471	13.018***	**0.492**	3.278**	0.362	5.645***	0.383	1.087ns	0.351	1.226ns	0.352
	Large	223	8.469***	0.474	11.833***	**0.575**	4.416***	0.364	6.357***	0.412	3.741***	0.350	1.683ns	0.318
	UTM	175	8.724***	0.520	12.499***	**0.635**	2.335*	0.334	5.837***	0.422	3.653***	0.359	0.399ns	0.314
	X Large	95	6.778***	0.714	10.624***	**0.807**	0.763ns	0.581	3.936***	0.634	2.808**	0.609	1.331ns	0.586
	Largest	34	5.593***	0.750	6.372***	**0.786**	0.776ns	0.539	3.575***	0.645	2.0214*	0.581	1.748ns	0.570

**Single predictor GLM (t-values) for invertebrates**	Small	1390	10.320***	0.071	12.810***	**0.105**	4.884***	0.016	8.866***	0.053	0.976ns	0.000	1.420ns	0.001
	Medium	570	7.550***	0.090	12.780***	**0.222**	4.019***	0.026	8.142***	0.103	1.335ns	0.001	0.323ns	-0.002
	Large	223	5.102***	0.101	7.990***	**0.221**	4.898***	0.094	7.789***	0.212	4.291***	0.073	-0.455ns	-0.004
	UTM	175	4.383***	0.095	6.382***	0.186	4.076***	0.082	6.504***	**0.192**	6.571***	0.195	-1.554ns	0.008
	X Large	95	4.408***	0.164	5.626***	0.246	4.684***	0.182	8.490***	**0.431**	6.089***	0.277	-1.199ns	0.005
	Largest	34	2.809***	0.173	2.038*	0.087	2.497*	0.137	5.509***	**0.471**	4.459***	0.364	-1.295ns	0.020
**Single predictor SLM (z-values) for invertebrates**	Small	1390	9.794***	**0.437**	9.147***	0.432	3.531***	0.403	3.777***	0.404	0.022ns	0.398	1.484ns	0.399
	Medium	570	7.442***	0.299	10.755***	**0.358**	3.506***	0.248	4.790***	0.260	0.099ns	0.231	0.290ns	0.232
	Large	223	4.978***	0.256	9.150***	**0.397**	5.482***	0.272	5.933***	0.278	3.020**	0.205	0.242ns	0.174
	UTM	175	3.961***	0.341	6.815***	**0.433**	2.718**	0.311	3.987***	0.339	3.935***	0.334	-0.443ns	0.283
	X Large	95	2.568*	0.644	7.468**	**0.760**	2.419*	0.642	3.397***	0.659	2.231*	0.638	0.239ns	0.620
	Largest	34	0.794ns	0.794	1.306ns	0.801	-0.006ns	0.791	3.039**	0.835	3.798***	**0.853**	-0.008ns	0.791
**Single predictor GLM (t-values) for habitat-forming species**	Small	1390	11.570***	0.087	13.400***	**0.114**	5.984***	0.024	11.870***	0.092	2.608**	0.004	0.397ns	-0.001
	Medium	570	9.745***	0.142	13.450***	**0.240**	4.427***	0.032	10.258***	0.155	2.955**	0.013	0.296ns	-0.002
	Large	223	6.213***	0.145	7.947***	0.219	4.580***	0.083	8.897***	**0.260**	6.492***	0.156	-0.621ns	-0.003
	UTM	175	6.763***	0.205	7.365***	0.234	3.455***	0.059	8.388***	**0.285**	8.246***	0.278	-1.137ns	0.002
	X Large	95	4.408***	0.164	5.897***	0.264	3.587***	0.112	8.467***	**0.429**	7.568***	0.375	-0.666ns	-0.006
	Largest	34	3.002**	0.195	2.493*	0.136	2.647*	0.154	5.505***	**0.470**	2.266***	0.540	-0.266ns	-0.029
**Single predictor SLM (z-values) for habitat-forming species**	Small	1390	10.079***	**0.550**	9.502***	0.540	3.631***	0.515	4.678***	0.518	0.252ns	0.510	1.929ns	0.511
	Medium	570	9.480***	0.408	11.359***	**0.440**	3.213**	0.327	5.686***	0.350	0.645ns	0.315	0.383ns	0.315
	Large	223	5.842***	0.350	9.214***	**0.455**	5.403***	0.337	6.041***	0.347	4.210***	0.299	0.026ns	0.250
	UTM	175	6.597***	0.513	8.033***	**0.556**	2.985**	0.422	5.824***	0.489	4.441***	0.445	-0.279ns	0.393
	X Large	95	2.971**	0.665	5.061***	**0.712**	1.208ns	0.640	2.839**	0.662	4.160***	0.688	-1.158ns	0.639
	Largest	34	0.109ns	0.423	0.802ns	0.433	0.683ns	0.430	3.264*	**0.529**	5.174***	0.677	0.111ns	0.424

The predictors are shallow water area (SW), coastline length (CL), habitat diversity based on area (HDIa), habitat diversity based on number of patches (HDIn), sea surface temperature (SST), and net primary productivity (NPP). Values under predictor variables are t-values for GLM and z-values for SLM. Asterisks indicate significance value of P: * (<0.05), ** (<0.01); *** (<0.001); ns (not significant). Sample size for each grid (n) is shown. The highest adjusted R^2 (GLM) and pseudo-R^2 (p-R^2; SLM) value within each grid size are highlighted in boldface.
