# Supplementary material for: Habitat Availability and Heterogeneity and the Indo-Pacific Warm Pool as Predictors of Marine Species Richness in the Tropical Indo-Pacific
Source: PLoS One. 2013 Feb 15;8(2):e56245. doi: 10.1371/journal.pone.0056245 (PMC3574161; doi:10.1371/journal.pone.0056245)
Supplement: Text S1 — Methods and results for UTM grids shifted to different centroid orientation. (PDF) [file pone.0056245.s018.pdf]

Text S1 Methods and results for UTM grids shifted to different centroid orientation.

To determine if the location of UTM centroids influences the result of the analysis, UTM grids were reoriented along a north-south plane ($UTM_{north/south}$), east-west plane ($UTM_{east/west}$), and northeast-southwest plane ($UTM_{northeast/southwest}$). Shifting the UTM does not show much difference with the results in the original UTM grid. The different UTM orientations also show the greatest concentration of SW in Arafura and Sunda Shelves (Figure S1a), while shifting the UTM grid north-south and northeast retains the highest coastline concentration in the Philippines, while shifting it east-west relocates this to the Bird's Head region in Papua, Indonesia (Figure S1b). Like the UTM grid, the highest HDIa index values are mostly found in the central Pacific Ocean, while the highest HDIn index values have more widespread locations across the Indo-Pacific (Figure S1c-d). The highest average SST an NPP values are found in similar locations as what the UTM grid displays (Figure S1e-f).

Shifting UTM grid centroids results in minor differences in the amount of variation explained. Analyses from GLM show that single-predictor models shallow water area, coastline length, HDIa, HDIn, and SST explain the amount of variation in species richness across all grids. Among these variables, CL explains the highest amount (Table S1a). Multiple-predictor GLM and SLM results to different combination of variables retain in the models, with R^2 and pseudo-R^2 values showing small changes among the different UTM grid orientations (Table S1b).
