# Supplementary material for: Habitat Availability and Heterogeneity and the Indo-Pacific Warm Pool as Predictors of Marine Species Richness in the Tropical Indo-Pacific
Source: PLoS One. 2013 Feb 15;8(2):e56245. doi: 10.1371/journal.pone.0056245 (PMC3574161; doi:10.1371/journal.pone.0056245)
Supplement: Text S2 — Sources for species range used in producing the distribution maps. (PDF) [file pone.0056245.s019.pdf]

Text S2 Sources for species range used in producing the distribution maps.

Pre-existing Geographical Information System (GIS) databases existed for marine invertebrates (corals [120], crustaceans, and molluscs [74]) and coral reef fishes [67]. For non-coral reef fishes, distribution information was collected from scientific articles [121-133], Food and Agriculture Organization field guides [134-136]; with additional literature too numerous to list but listed in Eschmeyer, 1998 [137] which was used to correct taxonomy and ranges, and expand ranges to the entire Indo-Pacific), species' catalogues [138-142], and an internet database [143]. Distributions of mangroves and seagrasses were taken from atlases [90,92]. Many of the maps were checked for accuracy and additional ones developed by experts as part of the International Union for Conservation of Nature, Global Marine Species Assessment [2]. In particular, distributions of reef-building corals, mangroves [100], seagrasses [101], and many families of coral-reef fishes were checked and further developed in this manner.

References

120. Veron JEN (2000) Corals of the world. Townsville: AIMS. 1382 p.

121. Trewavas E (1977) The sciaenid fishes (croakers or drums) of the Indo-West Pacific. Tran Zool Soc London 33: 253–541.

122. Vari RP (1978) The terapon perches (Percoidei, Teraponidae): A cladistic analysis and taxonomic revision. Bull Am Mus Nat Hist 159: 175–340.

123. Masuda H, Amaoka K, Araga C, Uyeno T, Yoshino T (1984) The fishes of the Japanese Archipelago. Tokyo, Japan: Tokai University Press. 437 p.

124. Heemstra PC (1986) Centracanthidae. In: Smith MM, Heemstra PC, eds. Smith's sea fishes. Berlin: Springer-Verlag. pp. 594–595.

125. Starnes WC (1988) Revision, phylogeny and biogeographic comments on the circumtropical marine percoid fish family Priacanthidae. Bull Mar Sci 43: 117–203(187).

126. Sasaki K, Amaoka K (1989) *Johnius distinctus* (Tanaka, 1916), a senior synonym of *J. tingi* (Tang, 1937) (Perciformes, Sciaenidae). Jap J Ichthyol 35: 466–468.

127. Sasaki K (1990) *Johnius grypotus* (Richardson, 1846), resurrection of a Chinese sciaenid species. Jap J Ichthyol 37: 224–229.

128. Sasaki K (1994) *Argyrosomus beccus*, a new sciaenid from South Africa. Jap J Ichthyol 41: 35–38.

129. Sasaki K (1995) A review of the Indo-West Pacific sciaenid genus *Panna* (Teleostei, Perciformes). Jap J Ichthyol 42: 27–37.

130. Sasaki K (1999) *Johnius (Johnieops) philippinus*, a new sciaenid from the Philippines, with a synopsis of species included in the subgenus Johnieops. Jap J Ichthyol 46: 271–279.

131. Talwar PK, Jhingran AG (1991) Inland fishes of India and adjacent countries. Vol. 2. Rotterdam: A. A. Balkema. 1158 p.

132. Yamashita T, Kimura S (2001) A new species, *Gazza squamiventralis*, from the east coast of Africa (Perciformes: Leiognathidae). Ichthyol Res 48: 161–166.

133. Iwatsuki Y, Kimura S, Yoshino T (2006) A new sparid, *Acanthopagrus akazakii*, from New Caledonia with notes on nominal species of *Acanthopagrus*. Ichthyol Res 53: 406–414.

134. Fischer W, Whitehead PJP (1974) FAO species identification sheets for fishery purposes: Eastern Indian Ocean (fishing area 57) and western central Pacific (fishing area 71). Rome: FAO.

135. Fischer W, Bianchi G (1984) FAO species identification sheets for fishery purposes: Western Indian Ocean (Fishing Area 51). Rome: FAO.

136. Carpenter KE, Niem VH (1998) FAO species identification guide for fisheries purposes: The living marine resources of the western central Pacific. Rome: FAO.

137. Eschmeyer WN (1998) Catalog of Fishes. San Francisco: California Academy of Sciences. 2905 p.

138. Collette BB, Nauen CE (1983) Scombrids of the world: An annotated and illustrated catalogue of tunas, mackerels, bonitos and related species known to date. Rome: FAO. 137 p.

139. Russell BC (1990) Nemipterid fishes of the world (threadfin breams, whiptail breams, monocle breams, dwarf monocle breams, and coral breams): Family Nemipteridae: An annotated and illustrated catalogue of nemipterid species known to date. Rome: FAO. 149 p.

140. Heemstra PC, Randall JE (1993) Groupers of the world (Family Serranidae, Subfamily Epinephelinae): An annotated and illustrated catalogue of the grouper, rock cod, hind, coral grouper and lyretail species known to date. Rome: FAO. 382 p.

141. Compagno LJV (2002) Sharks of the world: An annotated and illustrated catalogue of shark species known to date. Rome: FAO. 269 p.

142. Compagno LJV, Dando M, Fowler S (2005) Sharks of the world. Princeton: Princeton University Press. 480 p.

143. Robertson DR, Allen GR (2008) Shorefishes of the tropical Eastern Pacific online information system. Version 1.0. Smithsonian Tropical Research Institute. Available: http://biogeodb.stri.si.edu/sftep/intro1.php. Accessed 08 June 2006.
